# BRISC is required for optimal activation of NF-κB in Kupffer cells induced by LPS and contributes to acute liver injury

**DOI:** 10.1038/s41419-023-06268-z

**Published:** 2023-11-15

**Authors:** Wen Zhang, Kai Liu, Guang-Ming Ren, Yu Wang, Ting Wang, Xian Liu, Dong-Xu Li, Yang Xiao, Xu Chen, Ya-Ting Li, Yi-Qun Zhan, Shen-Si Xiang, Hui Chen, Hui-Ying Gao, Ke Zhao, Miao Yu, Chang-Hui Ge, Chang-Yan Li, Zhi-Qiang Ge, Xiao-Ming Yang, Rong-Hua Yin

**Affiliations:** 1 State Key Laboratory of Proteomics, Beijing Proteome Research Center, National Center for Protein Sciences (Beijing), Beijing Institute of Lifeomics, Beijing, 102206 China; 2 Department of Pharmaceutical Engineering, School of Chemical Engineering and Technology, Tianjin University, Tianjin, 300072 China; 3 Tianjin Key Laboratory of Food Science and Biotechnology, School of Biotechnology and Food Science, Tianjin University of Commerce, Tianjin, 300134 China; 4 Beijing Institute of Radiation Medicine, Beijing, 100850 China; 5 School of Basic Medical Sciences, Anhui Medical University, Hefei, 230032 Anhui Province China; 6 College of Life Science and Bioengineering, Faculty of Environmental and Life Sciences, Beijing University of Technology, Beijing, 100124 China; 7 Institute of Health Service and Transfusion Medicine, Beijing, 100850 China

**Keywords:** Acute inflammation, Ubiquitylation, Liver diseases

## Abstract

BRISC (BRCC3 isopeptidase complex) is a deubiquitinating enzyme that has been linked with inflammatory processes, but its role in liver diseases and the underlying mechanism are unknown. Here, we investigated the pathophysiological role of BRISC in acute liver failure using a mice model induced by D-galactosamine (D-GalN) plus lipopolysaccharide (LPS). We found that the expression of BRISC components was dramatically increased in kupffer cells (KCs) upon LPS treatment in vitro or by the injection of LPS in D-GalN-sensitized mice. D-GalN plus LPS-induced liver damage and mortality in global BRISC-null mice were markedly attenuated, which was accompanied by impaired hepatocyte death and hepatic inflammation response. Constantly, treatment with thiolutin, a potent BRISC inhibitor, remarkably alleviated D-GalN/LPS-induced liver injury in mice. By using bone marrow-reconstituted chimeric mice and cell-specific BRISC-deficient mice, we demonstrated that KCs are the key effector cells responsible for protection against D-GalN/LPS-induced liver injury in BRISC-deficient mice. Mechanistically, we found that hepatic and circulating levels of TNF-α, IL-6, MCP-1, and IL-1β, as well as TNF-α- and MCP-1-producing KCs, in BRISC-deleted mice were dramatically decreased as early as 1 h after D-GalN/LPS challenge, which occurred prior to the elevation of the liver injury markers. Moreover, LPS-induced proinflammatory cytokines production in KCs was significantly diminished by BRISC deficiency in vitro, which was accompanied by potently attenuated NF-κB activation. Restoration of NF-κB activation by two small molecular activators of NF-κB p65 effectively reversed the suppression of cytokines production in ABRO1-deficient KCs by LPS. In conclusion, BRISC is required for optimal activation of NF-κB-mediated proinflammatory cytokines production in LPS-treated KCs and contributes to acute liver injury. This study opens the possibility to develop new strategies for the inhibition of KCs-driven inflammation in liver diseases.

## Introduction

Acute liver failure (ALF) is a high-mortality disease that commonly occurs secondary to drug toxicity, infection, ischemia, or a devastating immune response [1, 2]. Rapid onset of massive hepatocyte apoptosis and necrosis is the main pathological feature of ALF [1, 3]. Accumulating evidence has demonstrated that proinflammatory cytokines, such as tumor necrosis factor (TNF)-α, interleukin (IL)-1, IL-6, and monocyte chemoattractant protein 1 (MCP-1), play critical roles in the occurrence and development of ALF, which together lead to hepatocyte death and liver failure [3]. Lipopolysaccharide (LPS), the endotoxins of gram-negative bacteria, is one of the most potent gut-derived stimulators of innate immune responses [4]. LPS is recognized by toll-like receptor 4 (TLR4) with the help of LPS-binding protein (LBP), cluster of differentiation 14 (CD14), and myeloid differentiation factor 2 (MD-2), which activates downstream signaling pathways, especially nuclear factor-κB (NF-kB) and mitogen-activated protein kinase (MAPK), and eventually leads to the release of proinflammatory mediators [4, 5]. LPS/TLR4 signaling is considered as a key pathway involved in both acute and chronic liver injury [4, 6]. Intraperitoneal administration of LPS alone induces a systemic inflammatory response that can result in death due to septic shock without triggering significant liver injury [7, 8]. However, administration of LPS in combination with other hepatotoxins, such as D-galactosamine (D-GalN), carbon tetrachloride, acetaminophen, and alcohol, aggravates hepatic inflammatory responses and necrosis, leading to ALF or acute-on-chronic liver failure (ACLF) in mice [6, 9, 10]. Hepatic resident macrophages, named Kupffer cells (KCs), are located within the hepatic sinusoids and seeded along sinusoidal endothelial cells, which act as a filter at the first line between the digestive tract and the liver [11, 12]. KCs thus are the first cell populations in the liver that respond to gut-derived LPS. In physiological conditions, the low quantity of gut-derived LPS is cleared mainly by KCs, leading to immune tolerance in the liver [13]. In both acute and chronic liver injury, portal venous levels of LPS are elevated due to increased intestinal permeability [4, 14]. The elevated LPS can activate KCs and cause the release of vast amounts of inflammatory mediators, resulting in hepatocytic cell death and recruitment of additional immune cells, such as neutrophils, monocytes, and natural killer (NK) cells, that further exacerbate liver injury [15]. Deletion of KCs in mice is reported to combat a variety of liver inflammatory diseases, such as D-GalN/LPS- or alcohol-induced liver injury, and diet-induced steatohepatitis [16–19]. Targeting KCs to block its proinflammatory activation thus is considered as a promising approach to treating liver diseases [20]. Increasing our understanding of the precise regulation of KCs activation by LPS, especially the production of proinflammatory mediators, may lead to the development of novel and more potent therapeutic strategies for human liver diseases.

BRISC (BRCC3 isopeptidase complex) is a deubiquitinating enzyme (DUB) that specifically cleaves K63-linked ubiquitin chains, which consists of four subunits including BRCC3, ABRO1, BRE, and MERIT40 [21]. ABRO1 and BRCC3 are the two most important components, as they control BRISC assembly and DUB activity [21]. BRCC3 is the catalytic subunit, but it alone displays no delectable cleavage activity unless complexed with ABRO1 in the cytoplasm [21, 22]. In addition to imparting DUB activity to BRCC3, ABRO1 serves as a scaffold protein that recruits the rest of the components to form the BRISC, and it is critical for maintaining the stability of BRISC complex. Many studies have shown that deficiency of ABRO1 substantially reduced the protein levels of the other components of BRISC, especially BRCC3 [23–25]. Previous studies have suggested that BRISC plays a critical role in regulating inflammation and antimicrobial immunity [23, 26–28]. BRISC-deficient mice that received intraperitoneal LPS injections display significantly reduced mortality, which may be attributed to decreased activation of type 1 interferon (IFN) signaling or NLRP3 inflammasome [23, 27]. BRISC complex cleaves the K63-linked polyubiquitin chains from actively engaged IFN receptor chain 1 (IFNAR1), thus limiting its internalization and lysosomal degradation. BRISC-deficient cells and mice exhibit attenuated responses to IFN and are protected from IFN-associated immunopathology [27]. In contrast, BRCC3 has been shown to inhibit type 1 IFN signaling by altering the ubiquitination of TRAF3 [29]. In response to LPS priming, BRISC is recruited to NLRP3 by ABRO1 and then deubiquitinates NLRP3 upon stimulation with activators, which leads to NLRP3 activation and the subsequent assembly of NLRP3 inflammasome. BRISC deficiency inhibits NLRP3-dependent secretion of IL-1β and IL-18, protecting against NLRP3-related inflammatory diseases in different mice models [23]. Moreover, pharmacological inhibition of BRISC alleviates NLRP3-driven diseases in mouse models including methionine-choline-deficient (MCD) diet-induced nonalcoholic fatty liver disease [30], which indicates a potential role of BRISC in liver diseases. Numerous studies have shown that NLRP3 inflammasome, as well as Type I interferon, plays an important role in various types of ALF caused by acetaminophen, viral hepatitis, liver ischemia-reperfusion, or other pathological reasons [31–34]. Nevertheless, whether and how BRISC participates in the pathological process of ALF is unknown.

Here, we investigated the pathophysiological role of BRISC in ALF using a mice model induced by D-galactosamine (D-GalN) plus LPS and primary cultured KCs. Our results demonstrated that LPS induced a rapid and dramatic elevation of BRISC components in KCs, prior to the occurrence of liver injury. Functionally, we found that global ablation of BRISC or specific deficiency of BRISC in myeloid cells and KCs but not in hepatocytes protected mice from D-GalN plus LPS-induced hepatotoxicity and mortality. Mechanistic studies revealed that BRISC deficiency suppressed LPS-induced NF-κB activation in KCs, thereby reducing the production of proinflammatory cytokines and alleviating liver injury.

## Results

### BRISC expression is induced in KCs by LPS and increased in the liver tissue of patients with ALF

**Fig. 1:**
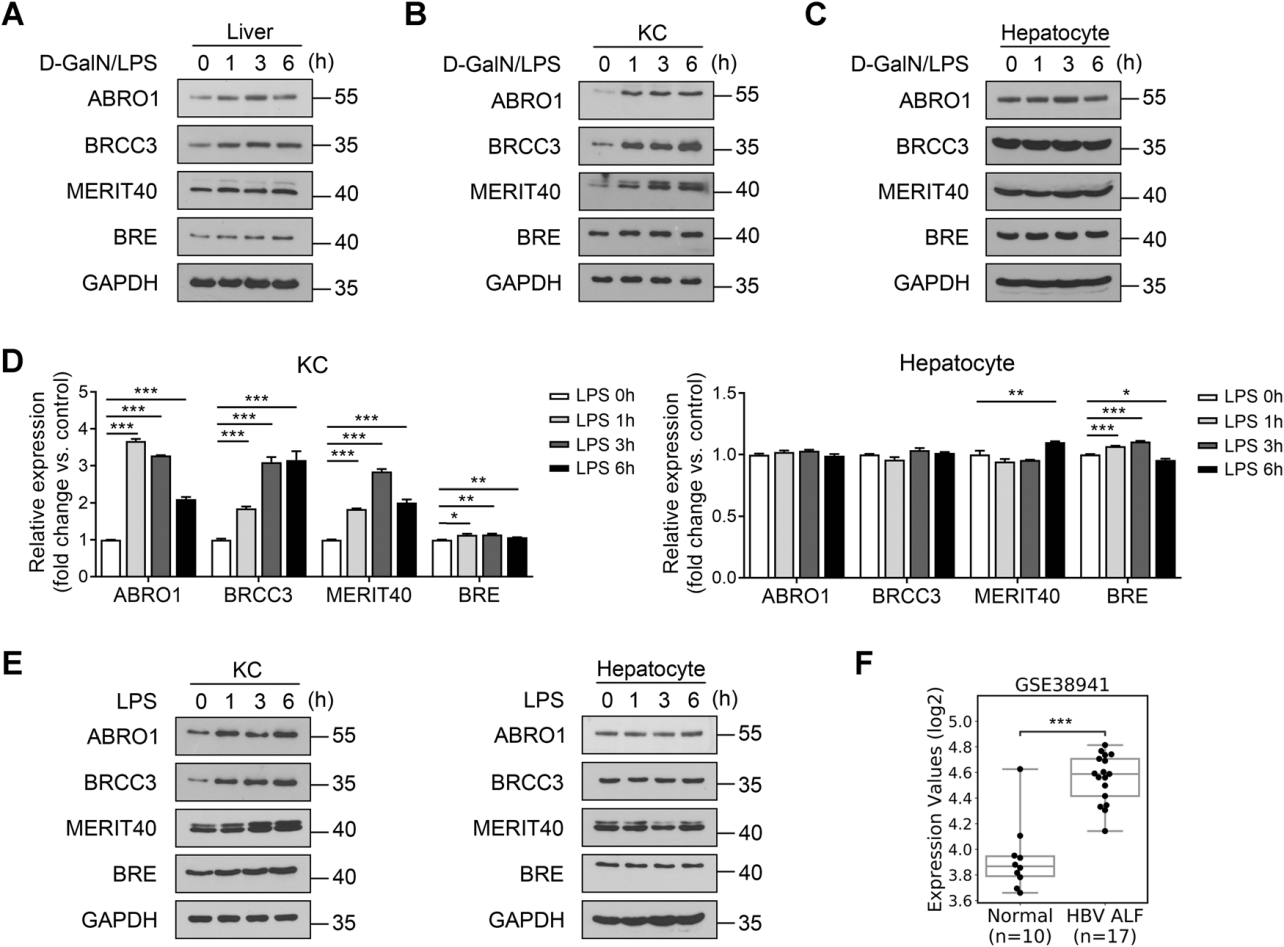
BRISC expression is induced in KCs by LPS and increased in the liver tissue of patients with ALF. WT mice were treated with a sublethal dose of D-GalN/LPS. Immunoblot analysis of BRISC components expression in **A** liver, **B** KCs, and **C** hepatocytes at the indicated times. KCs and hepatocytes were isolated and treated with LPS for various times. **D** Real-time PCR analysis and **E** immunoblot analysis of BRISC components expression at the indicated times. **F** BRCC3 expression level in the liver samples of patients with hepatitis B virus-associated ALF from published transcriptome dataset (GSE38941). Data are presented as mean ± SEM; Expression scores are shown as box plots, with the horizontal lines representing the median; the bottoms and tops of the boxes represent the 25th and 75th percentiles, respectively, and the vertical bars represent the range of data; *P < 0.05, **P < 0.01, ***P < 0.001; two-tailed unpaired *t*-test.

We first detected the alternation of protein levels of BRISC components, including ABRO1, BRCC3, MERIT40, and BRE, in the livers of mice challenged with D-GalN/LPS. The results showed that ABRO1 and BRCC3 were upregulated in mice livers as early as 1 h after D-GalN/LPS injection (Fig. 1A), which occurred prior to the elevation of the liver injury markers [35]. As hepatocytes and KCs are the main cells involved in D-GalN/LPS-induced liver injury [36, 37], we next detected BRISC expression in hepatocytes and KCs isolated from D-GalN/LPS-injected mice. We found that the protein levels of BRISC components were rapidly elevated in KCs but not hepatocytes upon D-GalN/LPS challenge (Fig. 1B, C). Furthermore, to evaluate whether LPS directly induces BRISC expression, KCs and hepatocytes were isolated from WT mice and then treated with LPS in vitro. By real-time PCR and western blot, both the mRNA and protein levels of BRISC components were increased by LPS in cultured KCs, but no substantial alterations were observed in hepatocytes (Fig. 1D, E, and Fig. S1A–C). To further assess the correlation of BRISC expression level with ALF, we analyzed the clinical data from the Gene Expression Omnibus (GEO) database, and observed a significant upregulation of BRCC3 mRNA in the liver samples of patients with hepatitis B virus-associated ALF (GEO data sets GSE38941) (Fig. 1F). These data suggest that the components of BRISC were directly induced in KCs upon LPS challenge and may be involved in the pathophysiological role of ALF.

### Deletion of BRISC potently protects mice from D-GalN/LPS-induced fatal hepatitis

**Fig. 2:**
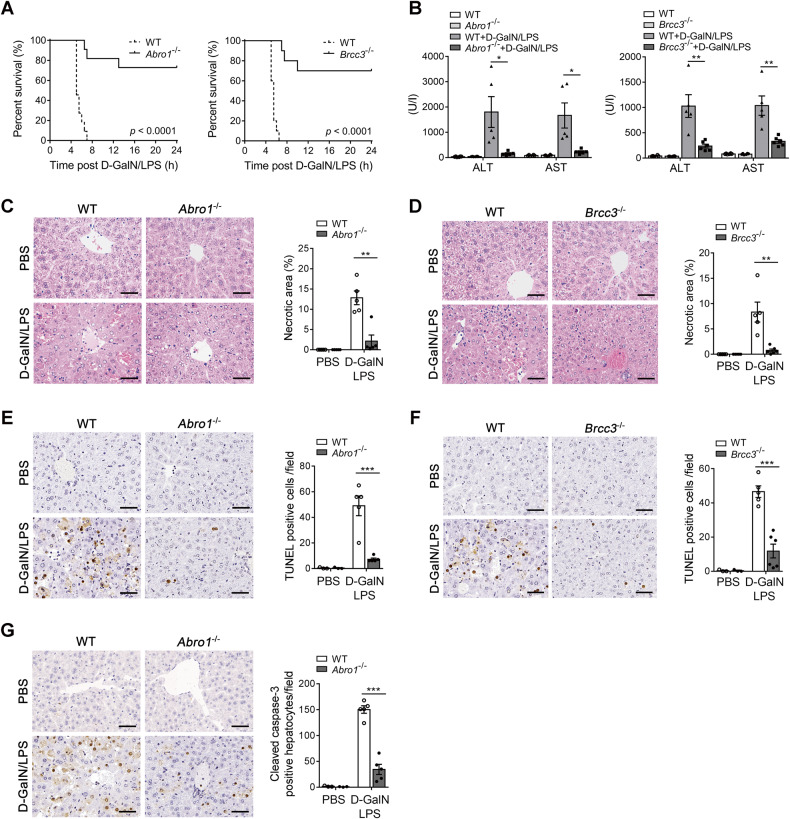
Deletion of BRISC potently protects mice from D-GalN/LPS-induced fatal hepatitis. **A** Survival rate of WT and *Abro1*^−/−^ mice or *Brcc3*^−/−^ mice after intraperitoneally injected with a lethal dose of D-GalN (700 mg/kg) plus LPS (15 μg/kg) (*N* = 10–11). Log-rank test. WT and *Abro1*^−/−^ mice or WT and *Brcc3*^−/−^ mice were treated with a sublethal dose of D-GalN (700 mg/kg) plus LPS (10 μg/kg) or PBS for 6 h (*N* = 3–6). **B** Serum levels of ALT and AST of WT and *Abro1*^−/−^ mice or *Brcc3*^−/−^ mice. Representative hematoxylin and eosin (H&E) staining of liver sections from **C** WT and *Abro1*^−/−^ mice or **D** WT and *Brcc3*^−/−^ mice. Necrotic area was shown as a percentage of the total field area. Representative images of TUNEL-stained liver sections of **E** WT and *Abro1*^−/−^ mice or **F** WT and *Brcc3*^−/−^ mice. TUNEL-positive cells per field were counted. **G** Representative liver sections of WT and *Abro1*^−/−^ mice that were stained with cleaved caspase-3. Cleaved caspase-3 positive hepatocytes in each field were counted. Scale bar, 50 μm. Data are presented as mean ± SEM; *P < 0.05, **P < 0.01, ***P < 0.001; two-tailed unpaired *t*-test.

To investigate the role of BRISC in LPS-induced inflammatory liver diseases, we established a mouse model of D-GalN/LPS-induced fatal hepatitis in global ABRO1 and BRCC3 knockout mice. A lethal dose of D-GalN/LPS treatment caused 100% mortality in WT mice within 8 h, whereas more than 70% *Abro1*^−/−^ and *Brcc3*^−/−^ mice were still alive and survived for long-term (Fig. 2A). Accordingly, serum levels of alanine aminotransferase (ALT) and aspartate aminotransferase (AST) in both *Abro1*^−/−^ and *Brcc3*^−/−^ mice were significantly lower than those in WT mice challenged with a sublethal dose of D-GalN/LPS at 6 h (Fig. 2B). Histological analysis confirmed reduced liver damage in both *Abro1*^−/−^ and *Brcc3*^−/−^ mice at 6 h after D-GalN/LPS injection compared to control animals, characterized by smaller areas of necrosis (Fig. 2C, D), decreased numbers of terminal deoxynucleotidyl transferase-mediated deoxyuridine triphosphate nick-end labeling (TUNEL) positive cells (Fig. 2E, F) and cleaved caspase-3 positive cells (Fig. 2G). These data indicate that eliminating BRISC has a strong impact on preventing hepatocyte death induced by D-GalN/LPS and thus significantly improves liver injury and mortality.

### BRISC deficiency impairs early proinflammatory cytokines production and inflammatory cell infiltration in the liver after D-GalN/LPS challenge

**Fig. 3:**
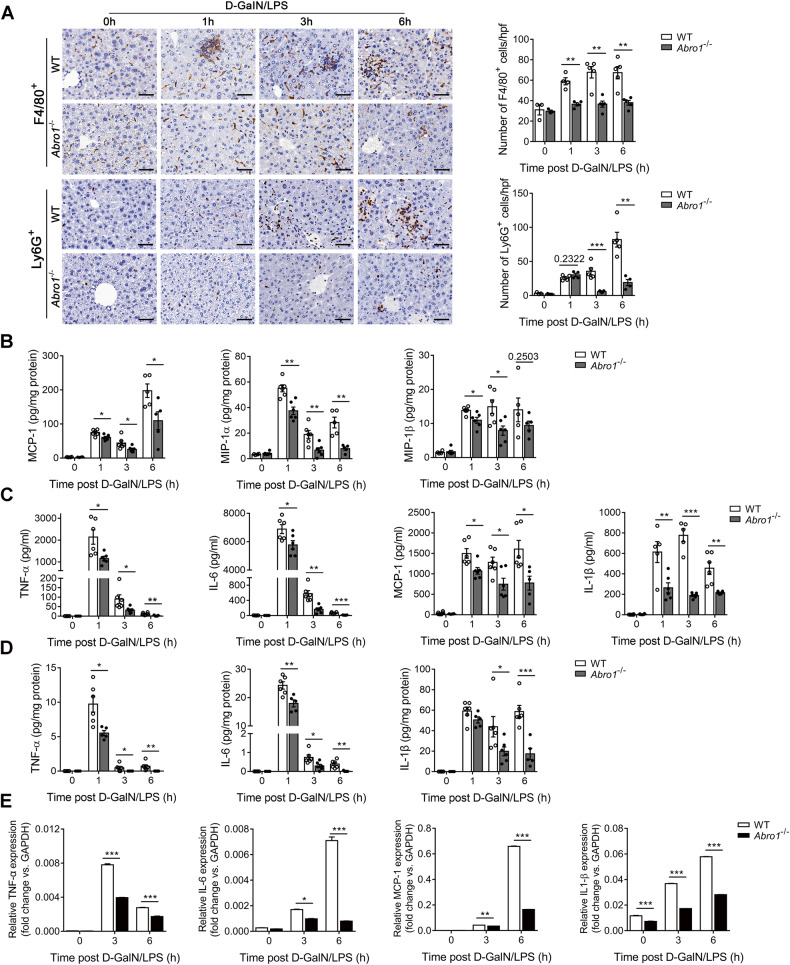
D-GalN/LPS-induced hepatic inflammation is attenuated in BRISC-deficient mice. WT and *Abro1*^−/−^ mice were treated with D-GalN/LPS for the indicated times (*N* = 3–6). **A** Immunohistochemistry (IHC) staining analysis of F4/80^+^ cells and Ly6G^+^ cells. Representative IHC pictures were shown and positive cells per high-power field (×400) were counted. Cytometric bead array (CBA) analysis of **B** the hepatic levels of MCP-1, MIP-1α, and MIP-1β, **C** the serum levels of TNF-α, IL-6, MCP-1, and IL-1β, and **D** the hepatic levels of TNF-α, IL-6, and IL-1β. **E** Relative mRNA levels of hepatic TNF-α, IL-6, MCP-1, and IL-1β. Scale bar, 50 μm. Data are presented as mean ± SEM; *P < 0.05, **P < 0.01, ***P < 0.001; two-tailed unpaired *t*-test.

Acute liver injury and fulminant hepatitis induced by D-GalN/LPS are associated with liver infiltration with remarkable immune cells [38]. We found that ABRO1 deficiency had no effect on the numbers of T lymphocytes (CD3^+^), macrophages (F4/80^+^), and neutrophils (Ly6G^+^) in the liver under physiological conditions (Fig. 3A and Fig. S2A). However, *Abro1*^−/−^ mice livers displayed significantly reduced numbers of F4/80^+^ cells as early as 1 h after D-GalN/LPS treatment (Fig. 3A). The infiltration of Ly6G^+^ cells in *Abro1*^−/−^ liver was markedly suppressed after 3 h of D-GalN/LPS treatment (Fig. 3A). While comparable CD3^+^ cells were observed between WT and *Abro1*^−/−^ livers either at 3 or 6 h after D-GalN/LPS treatment (Fig. S2A). Consistently, the livers of *Abro1*^−/−^ mice exhibited decreased levels of chemokines including MCP-1, MIP-1α, and MIP-1β compared to WT mice as early as 1 h after D-GalN/LPS treatment (Fig. 3B). The frequencies of major myelocyte and lymphocyte subpopulations in bone marrow (BM) and peripheral blood between *Abro1*^−/−^ and WT mice were comparable no matter before or after D-GalN/LPS challenge (Fig. S2B, C), which indicated that the suppression of intrahepatic leukocytes infiltration in *Abro1*^−/−^ mice were not due to altered BM responsiveness to LPS. Administration of D-GalN/LPS induced remarkable elevation of serum and liver TNF-α, IL-6, MCP-1, and IL-1β levels in WT mice, while these proinflammatory cytokines were significantly lower in the serum and liver of *Abro1*^−/−^ mice at all time points tested compared to WT mice (Fig. 3B–D). The serum and hepatic levels of TNF-α, the major factor that induced hepatotoxicity in D-GalN/LPS model [37], at 1 h following D-GalN/LPS treatment in *Abro1*^−/−^ mice were decreased by 48.5% and 45.5%, respectively (Fig. 3C, D). Similarly, a significant decrease in serum levels of proinflammatory cytokines was also observed in *Brcc3*^−/−^ mice challenged with D-GalN/LPS (Fig. S2D). In addition, the mRNA levels of TNF-α, IL-6, MCP-1, and IL-1β in the livers of *Abro1*^−/−^ mice were significantly decreased relative to WT mice after D-GalN/LPS treatment (Fig. 3E). These data indicate that deficiency of BRISC significantly attenuates early proinflammatory cytokines production and inflammatory cells infiltration in the liver after D-GalN/LPS challenge.

As hepatic macrophages and monocytes are the major cells to produce proinflammatory mediators leading to liver damage [39]. We thus examined cytokine production in hepatic KCs (F4/80^hi^CD11b^lo^), inflammatory monocytes (Mos, F4/80^int^CD11b^int^Ly6C^hi^), and monocyte-derived macrophages (MoMs, F4/80^int^CD11b^int^Ly6C^lo^) from WT and *Abro1*^−/−^ or *Brcc3*^−/−^ mice in response to D-GalN/LPS (Fig. S3A). The intracellular staining results revealed that the livers of *Abro1*^−/−^ and *Brcc3*^−/−^ mice displayed a remarkably lower percentage of TNF-α- and MCP-1-producing KCs but comparable percentages of TNF-α- and MCP-1-producing Mos and MoMs at 1 h after D-GalN/LPS injection (Fig. S3B, C), which indicates that BRISC deficiency selectively impairs production of proinflammatory cytokines in KCs at the early stage of D-GalN/LPS challenge in vivo.

### BRISC deficiency-mediated hepatoprotective effect is dependent on hematopoietic cells

**Fig. 4:**
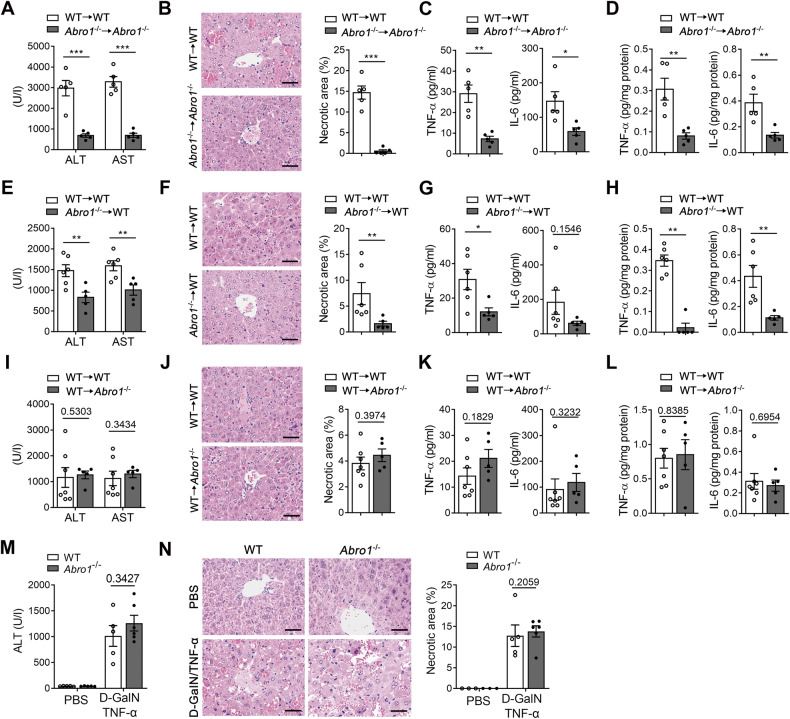
BRISC deficiency-mediated hepatoprotective effect is dependent on hematopoietic cells. BM chimeras were generated by BM transplantation with depletion of KCs prior to irradiation. **A**–**D** BM cells were transplanted from WT mice (CD45.2) to WT mice (CD45.2) or from *Abro1*^−/−^ mice (CD45.2) to *Abro1*^−/−^ mice (CD45.2) (*N* = 5). **E**–**H** BM cells from WT mice (CD45.2) or *Abro1*^−/−^ mice (CD45.2) were transplanted into WT mice (CD45.1) (*N* = 5–6). **I**–**L** BM cells from WT mice (CD45.1) were transplanted into WT mice (CD45.2) or *Abro1*^−/−^ mice (CD45.2) (*N* = 5–7). The mice were treated with D-GalN/LPS 10 weeks after transplantation and the liver injury was examined at 6 h after D-GalN/LPS injection. **A**, **E**, **I** Serum levels of ALT and AST. **B**, **F**, **J** Representative H&E staining and percentage of necrotic area of liver sections. CBA analysis of the **C**, **G**, **K** serum and **D**, **H**, **L** hepatic levels of TNF-α and IL-6. WT and *Abro1*^−/−^ mice were treated with D-GalN/TNF-α or PBS for 6 h (*N* = 3–6). Liver injury was evaluated by **M** serum ALT level and **N** H&E staining 6 h after D-GalN/LPS injection. Necrotic area was shown as a percentage of the total field area. Scale bar, 50 μm. Data are presented as mean ± SEM; *P < 0.05, **P < 0.01, ***P < 0.001; two-tailed unpaired *t*-test.

To detect the responsible cell types for the amelioration of D-GalN/LPS-induced liver injury in BRISC-deficient mice, we generated BM-reconstituted chimeric mice using a combination of clodronate liposomes-mediated KCs depletion, irradiation and BM-transplantation (BMT) [40]. This protocol achieved full BM engraftment and KCs replenishment. By flow cytometry analysis of leukocytes from peripheral blood and livers of recipient mice 8 weeks after transplantation, we determined that ABRO1 deficiency had no significant effects on hematopoietic engraftment and multi-lineage reconstitution (Fig. S4A–D). In agreement with our above results, *Abro1*^−/−^ mice (CD45.2) transplanted with *Abro1*^−/−^ BM (CD45.2) displayed markedly reduced liver injury compared to WT mice (CD45.2) transplanted with WT BM (CD45.2) after D-GalN/LPS challenge, as assessed by measuring serum aminotransferase and liver necrotic area, as well as serum and hepatic proinflammatory cytokines (Fig. 4A–D). Notably, significantly attenuated liver injury induced by D-GalN/LPS was observed in WT mice (CD45.1) receiving *Abro1*^−/−^ BM (CD45.2) relative to WT mice (CD45.1) receiving WT BM (CD45.2) (Fig. 4E–H), indicating that hematopoietic cells lacking ABRO1 contribute to the hepatoprotective efficacy of *Abro1*^−/−^ mice. In contrast, D-GalN/LPS-induced liver injury in *Abro1*^−/−^ recipients (CD45.2) of WT BM (CD45.1) was not significantly different from in WT recipients (CD45.2) of WT BM (CD45.1) (Fig. 4I–L), suggesting that non-hematopoietic cells null of ABRO1 are dispensable for the hepatoprotective efficacy of *Abro1*^−/−^ mice.

As BRISC deficiency resulted in reduced hepatocyte death induced by D-GalN/LPS, we next investigated whether BRISC affects hepatocyte response to TNF-α. In isolated primary hepatocytes, lack of ABRO1 did not potently reduce D-GalN/TNF-α induced hepatotoxicity (Fig. S5A) and TNF-α induced downstream signaling pathway activation (Fig. S5B). Consistently, *Abro1*^−/−^ mice lost the ability to combat liver damage induced by direct administration of TNF-α to D-GalN-sensitized mice (Fig. 4M, N). These data imply that hepatocytes appear to have a limited role in protecting against D-GalN/LPS-induced liver injury in *Abro1*^−/−^ mice.

### Kupffer cells contribute to the protection of BRISC-deficient mice from LPS-induced liver injury

**Fig. 5:**
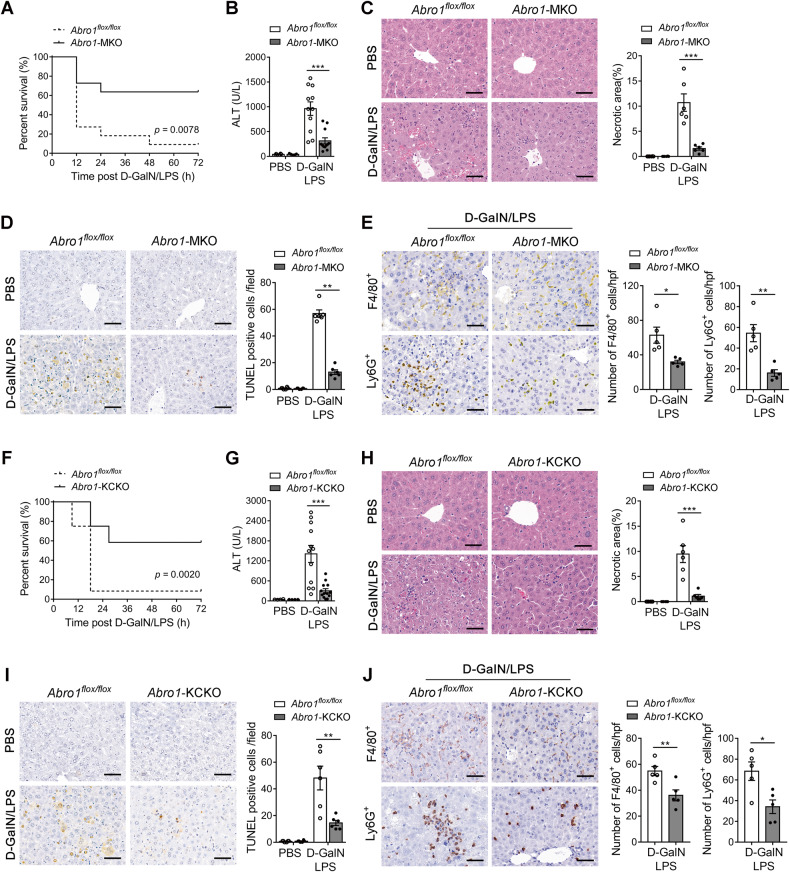
Kupffer cells contribute to the protection of BRISC-deficient mice from LPS-induced liver injury. **A** Survival curve for *Abro1*^flox/flox^ and *Abro1*-MKO mice challenged with a lethal dose of D-GalN/LPS (*N* = 11). Log-rank test. *Abro1*^flox/flox^ and *Abro1*-MKO mice were treated with a sublethal dose of D-GalN/LPS for 6 h (*N* = 5–12). Liver injury was evaluated by **B** serum ALT level and **C** H&E staining. **D** Hepatocyte apoptosis was evaluated by TUNEL staining. **E** Hepatic inflammatory cells infiltration was measured by F4/80^+^ and Ly6G^+^ immunohistochemistry staining. **F** Survival curves for *Abro1*^flox/flox^ and *Abro1*-KCKO mice challenged with a lethal dose of D-GalN/LPS (*N* = 12). Log-rank test. *Abro1*^flox/flox^ and *Abro1*-KCKO mice were treated with a sublethal dose of D-GalN/LPS for 6 h (*N* = 4–13). **G** Serum ALT and **H** H&E staining 6 h after D-GalN/LPS injection. Necrotic area was shown as a percentage of the total field area. **I** Hepatocyte apoptosis was evaluated by TUNEL staining. **J** Hepatic inflammatory cells infiltration was measured by F4/80^+^ and Ly6G^+^ immunohistochemistry staining. Representative images were shown and the number of positive cells in each high-power field was counted. Scale bar, 50 μm. Data are presented as mean ± SEM; *P < 0.05, **P < 0.01, ***P < 0.001; two-tailed unpaired *t*-test.

To determine which cell populations contribute to BRISC deficiency-dependent protection against D-GalN/LPS-induced liver injury, we generated *Abro1*^flox/flox^ mice (Fig. S6A) and crossed them with *Alb-Cre, Lyz2-Cre*, or *Clec4f-Cre* mice [41] to get mice with specific ABRO1 deletion on hepatocytes (designated *Abro1*-HKO), myeloid cells (designated *Abro1*-MKO), and KCs (designated *Abro1*-KCKO). The genotypes of these mice were verified by western blot (Fig. S6B–D). The protein levels of the other components of BRISC complex in ABRO1-deicient cells were also examined and showed substantial reduction in all the five cell types tested (Fig. S6E), indicating an essential role of ABRO1 in maintaining BRISC stability, which is consistent with previous reports [23–25]. These mice were then subjected to D-GalN/LPS challenge. The results showed that myeloid-specific and KC-specific deletion of ABRO1 led to a profound phenotype similar to global knockout of ABRO1 in the model of D-GalN/LPS-induced fetal hepatitis, as manifested by elevated survival rate, decreased serum ALT, reduced liver necrotic area and hepatocyte apoptosis, and less macrophages and neutrophils infiltration compared to *Abro1*^flox/flox^ mice (Fig. 5A–J). In contrast, no significant improvement in liver injury was observed in D-GalN/LPS-treated *Abro1*-HKO mice compared to control mice (Fig. S7A, B). Overall, these data indicate that KCs are critical for the protection against D-GalN/LPS-induced liver injury in BRISC-deficient mice. Notably, the percentage and number of KCs in *Abro1*^−/−^ mice were comparable with WT control mice (Fig. S8A). *Abro1*^−/−^ KCs showed normal expression of common KCs’ markers and M1/M2 polarization markers (Fig. S8B, C). Therefore, BRISC deficiency combat D-GalN/LPS-induced liver injury may not be due to the effect on KCs development under steady-state.

### BRISC deficiency selectively suppresses LPS-induced proinflammatory cytokines production in KCs in vitro

**Fig. 6:**
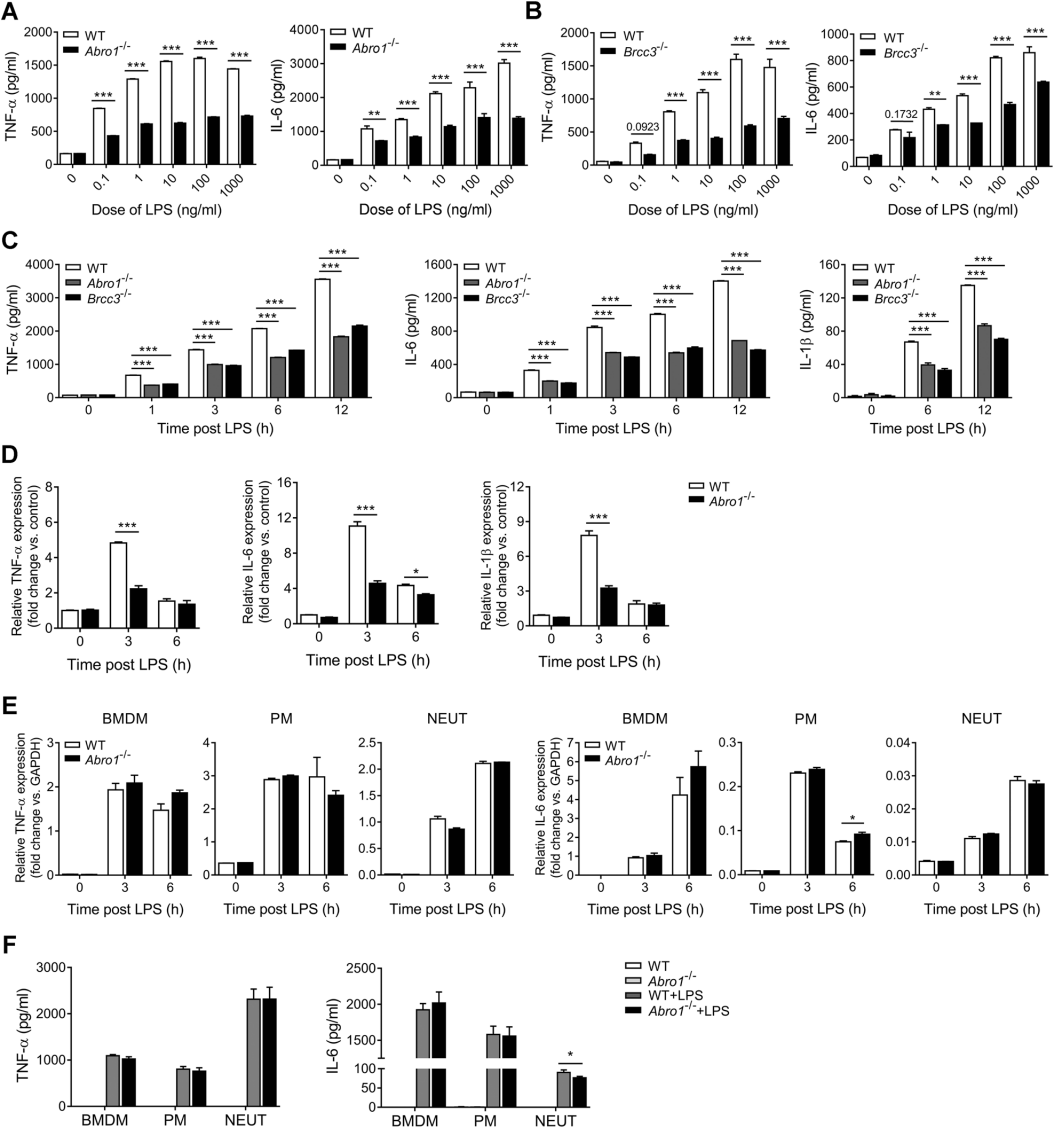
BRISC deficiency selectively suppresses LPS-induced proinflammatory cytokines production in KCs in vitro. CBA analysis of TNF-α and IL-6 from **A** WT and *Abro1*^−/−^ or **B** WT and *Brcc3*^−/−^ KCs stimulated with various doses of LPS for 3 h. **C** CBA analysis of TNF-α, IL-6, and IL-1β from WT, *Abro1*^−/−^, and *Brcc3*^−/−^ KCs stimulated with 100 ng/ml LPS for the indicated times. **D** Relative mRNA levels of TNF-α, IL-6, and IL-1β in WT and *Abro1*^−/−^ KCs treated with 100 ng/ml LPS for the indicated times. **E** Relative mRNA levels of TNF-α and IL-6 in WT and *Abro1*^−/−^ BMDMs, PMs, and NEUTs stimulated with 100 ng/ml LPS for the indicated times. **F** CBA analysis of TNF-α and IL-6 from WT and *Abro1*^−/−^ BMDMs, PMs, and neutrophils stimulated with 100 ng/ml LPS for 12 h. Data are presented as mean ± SEM; *P < 0.05, **P < 0.01; ***P < 0.001; two-way ANOVA with Bonferroni’s multiple comparisons test (**A**–**E**) or two-tailed unpaired *t*-test (**F**).

To confirm the pivotal role of BRISC in KCs proinflammatory activation, we next examined the effect of BRISC deficiency on the production of LPS-induced proinflammatory cytokines in KCs in vitro. The results showed that *Abro1*^−/−^ KCs, as well as *Brcc3*^−/−^ KCs, produced far less TNF-α and IL-6 than WT cells throughout the entire dose range of LPS (0.1 ng/ml to 1 μg/ml) (Fig. 6A, B). Consistently, no matter the lack of ABRO1 or BRCC3 significantly inhibited TNF-α, IL-6, and IL-1β production in KCs stimulated with 100 ng/ml LPS for various times (Fig. 6C). The observations that ABRO1 deficiency almost had no effects on the viability of KCs treated with LPS suggested the suppression of cytokines production was not due to reduced number of cells (Fig. S9A). Furthermore, we found that the LPS-induced upregulation of TNF-α, IL-6, and IL-1β mRNA levels was markedly attenuated in *Abro1*^−/−^ KCs compared with WT cells (Fig. 6D), suggesting that BRISC suppresses proinflammatory cytokines production in KCs through decreasing their transcription, which is different from our previous observation in LPS-treated bone marrow-derived macrophages (BMDMs) [23]. A previous observation showed that IL-1β mRNA was decreased in blood leukocytes of ABRO1 knockout mice after LPS challenge [27], suggesting that BRISC may differentially modulate the inflammatory response to LPS stimulation in different cell types. We thus investigated whether ABRO1 regulates KCs response to LPS in a cell-specific manner. BMDMs, resident peritoneal macrophages (PMs), and BM-derived neutrophils (NEUTs) were isolated from *Abro1*^−/−^ and WT mice, and then stimulated with LPS. The mRNA levels and the release of TNF-α and IL-6 in all of the three types of *Abro1*^−/−^ cells were comparable with control groups (Fig. 6E, F), suggesting that BRISC deletion suppresses LPS-induced proinflammatory cytokines production in a cell-specific manner.

### BRISC positively regulates NF-κB activation in LPS-stimulated KCs

**Fig. 7:**
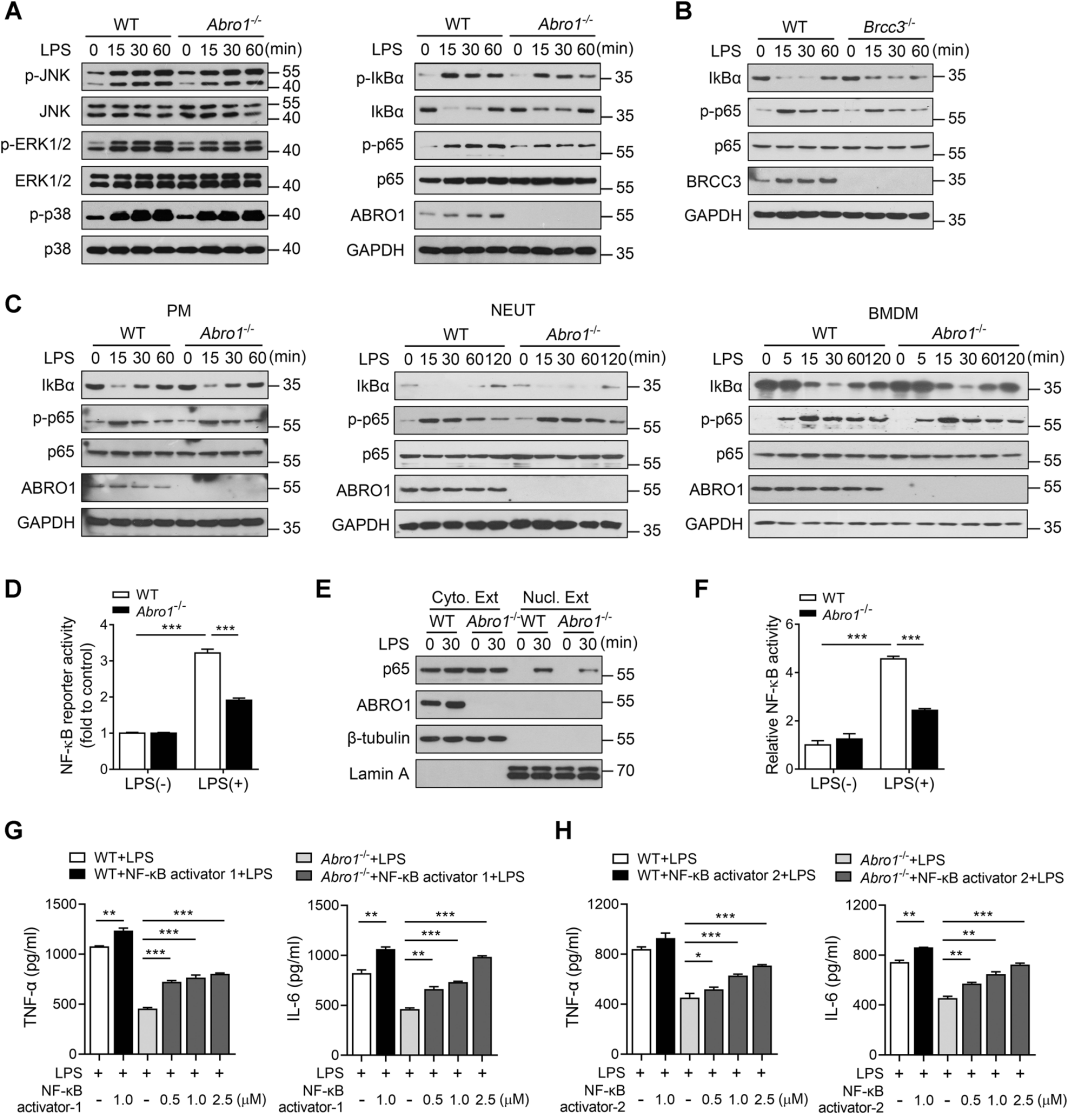
BRISC positively regulates NF-κB activation in LPS-stimulated KCs. **A** WT and *Abro1*^−/−^ KCs were stimulated with 100 ng/ml LPS for various times. Immunoblot analysis of the indicated target proteins. **B** WT and *Brcc3*^−/−^ KCs were stimulated with 100 ng/ml LPS for various times, followed by immunoblot analysis of the indicated target proteins. **C** WT and *Abro1*^−/−^ PMs, neutrophils, and BMDMs were stimulated with 100 ng/ml LPS for the indicated time points, followed by immunoblot analysis of the indicated target proteins. **D** WT and *Abro1*^−/−^ KCs transduced with lentivirus expressing NF-κB-luciferase reporter gene were treated with 100 ng/ml LPS for 3 h. The luminescence levels were measured and normalized to control values. Nuclear and cytoplasmic proteins of WT and *Abro1*^−/−^ KCs were extracted after stimulation with LPS for 30 min. **E** Immunoblot analysis of p65 expression in the cytoplasm and nucleus. **F** ELISA of the DNA binding activity of nuclear NF-κB p65. WT and *Abro1*^−/−^ KCs were pre-treated with NF-κΒ activator 1 **G** or NF-κΒ activator 2 **H** for 6 h and then treated with 100 ng/ml LPS for 3 h. CBA analysis of TNF-α and IL-6. Data are presented as means ± SEM; **P < 0.01; ***P < 0.001; two-tailed unpaired *t*-test.

We further tested the activation of the downstream pathways of LPS/TLR4 signaling in BRISC-deficient cells and found that the phosphorylation of JNK, ERK1/2, and p38 MAP kinase was unaffected in KCs lacking ABRO1, but the phosphorylation and degradation of IκBα, as well as the phosphorylation of p65, were inhibited (Fig. 7A). *Brcc3*^−/−^ KCs stimulated with LPS also showed decreased degradation of IκBα and phosphorylation of p65 as compared to WT cells (Fig. 7B). In contrast, ABRO1- deficient PMs, NEUTs, and BMDMs had similar kinetics of IκBα degradation and p65 phosphorylation induced by LPS (Fig. 7C), which reconfirmed that BRISC may regulate LPS-induced inflammation response in a cell type-dependent manner. When *Abro1*^−/−^ KCs were infected with lentivirus expressing NF-κB-luciferase reporter gene, LPS-induced luciferase activity was significantly lower than that in control cells (Fig. 7D). Moreover, lack of ABRO1 in KCs significantly reduced LPS-induced p65 nuclear translocation and NF-κB DNA binding activity (Fig. 7E, F). We next investigated whether it is possible to rescue the deficits of BRISC-deficient KCs by the utilize of two small molecule activators of p65, NF-κB activator 1 and NF-κB activator 2, which were reported to up-regulate NF-κB p65 transcriptional activity [42]. As expected, activation of NF-κB pathway markedly reversed the suppression of cytokines production in ABRO1-deficient KCs upon LPS treatment (Fig. 7G, H). Together with the results that BRISC deficiency resulted in decreased expression of LPS-induced NF-κB target genes including TNF-α, IL-6, and IL-1β, our findings indicated that BRISC positively regulates LPS-induced activation of the NF-κB signaling pathway in KCs.

### Pharmacological targeting of BRISC attenuates D-GalN/LPS-induced liver injury

**Fig. 8:**
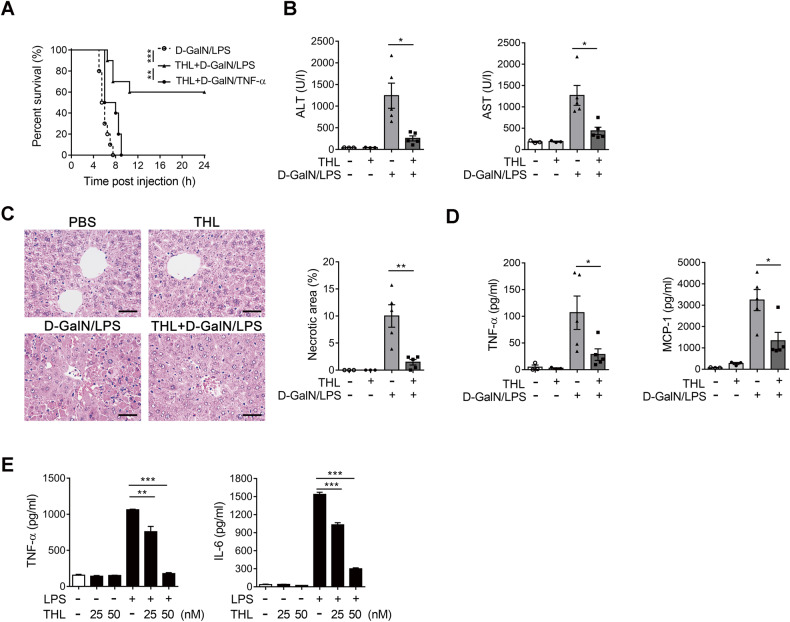
Pharmacological targeting of BRISC attenuates D-GalN/LPS-induced liver injury. **A** Survival curves for WT and *Abro1*^−/−^ mice challenged with a lethal dose of D-GalN/LPS or D-GalN/TNF-α (*N* = 10). Log-rank test. WT mice received two intraperitoneal injections of THL (2.5 mg/kg) 1 h before and 1 h after PBS or a sublethal dose of D-GalN/LPS administration (*N* = 3–5). **B** Serum levels of ALT and AST, **C** representative H&E staining and percentage of necrotic area of liver sections 6 h after D-GalN/LPS injection. **D** CBA analysis of the serum levels of TNF-α and MCP-1. **E** WT KCs pre-treated with 50 nM THL or vehicle control for 2 h were left unstimulated or stimulated with 1 μg/ml LPS for 6 h. CBA analysis of TNF-α and IL-6. Scale bar, 50 μm. Data are presented as mean ± SEM; *P < 0.05, **P < 0.01; ***P < 0.001; two-tailed unpaired *t*-test.

We next tested whether pharmacological targeting of BRISC protects mice against D-GalN/LPS-induced liver injury, and found that treating with thiolutin (THL), a potent inhibitor of BRISC [30, 43], markedly increased mice survival after injection of a lethal dose of D-GalN/LPS (Fig. 8A). Interestingly, 15 μg/kg TNF-α challenge in D-GalN-sensitized mice almost completely diminished the beneficial effect conferred by THL treatment on mice survival (Fig. 8A), indicating suppression of TNF-α production is essential for the hepatoprotection by THL against D-GalN/LPS-induced liver injury. Mice treated with THL displayed significantly attenuated liver injury induced by D-GalN/LPS, as shown by much lower serum ALT and AST as well as far less liver necrotic area (Fig. 8B, C). THL treatment also decreased the production of serum TNF-α and MCP-1 in mice challenged with D-GalN/LPS (Fig. 8D). In addition, LPS-induced TNF-α and IL-6 release in ex vivo cultured KCs were remarkably inhibited by THL (Fig. 8E). Notably, THL treatment almost had no effect on the viability of KCs stimulated with LPS (Fig. S9B). These data suggest that targeting BRISC with small molecular inhibitors impairs KCs-mediated cytokines production in response to LPS and thereby attenuates D-GalN/LPS-induced liver injury.

## Discussion

In the present study, we found that BRISC components including ABRO1 and BRCC3 in KCs were rapidly induced upon LPS treatment in vitro or by the injection of LPS in D-GalN-sensitized mice. Global deficiency of BRISC results in effective protection of mice against D-GalN/LPS-induced acute liver injury. Further, we demonstrated that KCs serve as the primary effector cells responsible for the hepatoprotective effects observed in BRISC-deficient mice by utilization of bone marrow reconstituted chimeric mice and hepatocyte-, myeloid-, and KC-specific ABRO1-deficient mice. We obtained several lines of evidence demonstrating that BRISC is involved in the early pathophysiological process of D-GalN/LPS-induced liver damage by promoting inflammatory factors production in KCs. First, D-GalN/LPS-induced liver damage occurs after the dramatic elevation of BRISC expression in KCs. Second, both circulating and hepatic levels of proinflammatory cytokines are markedly attenuated in BRISC-deficient mice as early as 1 h after D-GalN/LPS challenge. In particular, circulating and hepatic levels of TNF-α, a major driving force of D-GalN/LPS-induced liver injury, are approximately 45% lower in *Abro1*^−/−^ mice than in control mice at this time. Third, BRISC-deficient KCs, but not hepatic inflammatory monocytes and monocyte-derived macrophages, have significantly lower cytokines production at 1 h after D-GalN/LPS treatment. Fourth, KC-specific ABRO1-deficient mice are resistant to D-GalN/LPS-induced liver injury, which is accompanied by a weakened production of proinflammatory cytokines. Fifth, BRISC-deficient KCs have a defect in proinflammatory cytokines production in response to LPS in vitro. These data suggest that the expression of BRISC in KCs is rapidly induced by LPS, which acts as a signal to promote LPS-induced proinflammatory cytokines production in KCs and then contributes to D-GalN/LPS-induced liver injury. To the best of our knowledge, this work for the first time links BRISC to ALF and provides new insight into our understanding of the regulation of proinflammatory cytokines production in KCs in response to LPS. The upregulated expression of BRCC3 mRNA in the liver samples of patients with hepatitis B virus-associated ALF seems to imply that increased BRISC activation is associated with the development of human ALF. LPS-induced proinflammatory cytokines production of KCs plays a central role in initiating and driving liver inflammation, which is deemed to contribute to various acute and chronic liver diseases [15]. Whether BRISC regulates LPS-induced release of inflammatory cytokines in KCs is also involved in the pathogenesis of other liver diseases such as liver fibrosis is of interest. Selective inhibition of KCs proinflammatory activation has been considered as an effective strategy for the treatment of liver diseases [20]. Our findings provide a candidate target for selective inhibition of LPS-mediated inflammatory activation of KCs. Furthermore, BRISC is also essential for the optimal activation of IFNAR1 and NLRP3 inflammasome pathways in hepatic macrophages, both of which are involved in the pathogenesis of a wide variety of liver diseases [23, 27]. Therefore, targeting BRISC could inhibit multiple inflammatory signaling pathways in the liver and may have beneficial effects on the development of liver diseases.

Upon LPS treatment, LPS/TLR4 signaling promptly induces potent innate immune responses that signal through myeloid differentiation factor 88 (MyD88)-dependent and TIR-domain-containing adapter-inducing interferon-β (TRIF)-dependent pathways to activate NF-κB, activator protein 1 (AP-1), and interferon regulatory factors (IRFs), which then lead to the expression and release of vast amounts of inflammatory mediators [44]. We demonstrated that the activation of JNK, ERK1/2, and p38 MAP kinase induced by LPS was unaffected in KCs lacking ABRO1, but the NF-κB pathway was attenuated, as evidenced by the decrease of IκBα degradation, p65 phosphorylation, NF-κB-luciferase reporter activity, p65 nuclear translocation, as well as NF-κB DNA binding activity. Although several transcription factors have been implicated in LPS/TLR4-induced inflammatory cytokines gene expression in KCs, the transcription factor NF-κB is considered to play a central role [45–47]. By using commercially available NF-κB activators, we further verified that the specific activation of the NF-κB pathway exhibited a significant restorative effect on the suppressed production of cytokines in ABRO1-deficient KCs upon LPS treatment. Our findings thus indicate that BRISC deletion diminished LPS-induced inflammatory cytokines production in KCs by impairment of LPS-triggered NF-κB activation. Interestingly, BMDMs, peritoneal macrophages, and neutrophils from BRISC-deficient mice had normal NF-κB activation and cytokines production in response to LPS, suggesting BRISC, as transforming growth factor β-activated kinase 1 (TAK1) and haem-oxidized IRP2 ubiquitin ligase 1 (HOIL-1) reported previously [48, 49], plays a cell type-specific role in regulation of LPS-induced NF-κB activation. However, the molecular mechanism by which BRISC selectively regulates LPS-induced NF-κB activation in KCs remains unrevealed. The reasons may include the fact that the responsiveness of KCs to LPS/TLR4 is different from other cells with a more complex and precise regulatory network [50, 51], and that macrophages exhibit a large degree of intrinsic functional heterogeneity [52, 53]. This study also raised a question of how the deubiquitination activity of BRISC is linked to its promotive effect on NF-κB activation in KCs. A significant amount of research has described that K63-linked polyubiquitin modification plays a positive role in the activation of NF-κB pathway, thus K63-specific DUBs always negatively regulate NF-κB pathway [54]. The observation that BRISC functions as a K63-specific DUB but has a positive role in the regulation of TLR4-NF-κB signaling pathway in KCs indicates that BRISC is unlikely to directly target the classic K63-linked polyubiquitinated proteins in NF-κB pathway, such as TNF receptor-associated factor 6 (TRAF6) and NF-κB essential modulator (NEMO). Instead, it may target the regulators of these multiubiquitinated proteins. To reveal the precise mechanism by which BRISC selectively regulates LPS/TLR4-induced NF-κB activation in KCs may help us to understand the cell-specific mechanism of NF-κB activation.

In summary, this study demonstrated that the drastic elevation of BRISC expression in KCs in response to LPS is an early and obligatory step for LPS-induced liver injury in D-GalN-sensitized mice by triggering NF-κB-mediated production of proinflammatory cytokines. Our findings open the possibility to develop new strategies for the inhibition of KCs-driven inflammation in liver diseases.

## Materials and methods

### Mice

*Abro1*^−/−^ and *Brcc3*^−/−^ mice have been generated as previously described [23]. CD45.1^+^ mice on a C57BL/6 background were kindly provided by Tao Cheng (State Key Laboratory of Experimental Hematology, Institute of Hematology & Blood Diseases Hospital, Tianjin, China). *Abro1*^flox/+^ mice were generated by Shanghai Model Organisms Center Inc using CRISPR/Cas9-mediated genome editing on a C57/BL/6J background. *Clec4f-Cre* mice were gifted by Dr. Li Tang (Beijing Institute of Lifeomics) [41]. *Lyz2-Cre* mice [The Jackson Laboratory, 004781, B6.129P2-Lyz2^tm1(cre)Ifo^/J] and *Alb-Cre* mice [NM-KI-00002, B6.129S-*Alb*^tm1.1(CreERT2)Smoc^] were kindly provided by Shanghai Model Organisms Center Inc. In all experiments, genetically modified mice were systematically compared to their sex-, age-, and weight-matched controls, and randomly allocated to different experimental groups. All mice were maintained in individually ventilated cages under specific pathogen-free conditions at the animal facility of the Laboratory Animal Center of Beijing Institute of Lifeomics with a 12 h light-dark cycle and allowed free access to food and water. All animal experiments were reviewed and approved by the Institutional Animal Care and Use Committee of Beijing Institute of Lifeomics. Male and female mice between 8 and 10 weeks of age were used in the studies.

### Acute liver injury mouse model

Liver injury was typically induced by intraperitoneal injections of 700 mg/kg D-GalN (Sigma, G0500), followed by 10 μg/kg LPS (Sigma, L6529) or 10 μg/kg TNF-α (Peprotech, AF-315-01A). For survival analysis, a lethal dose of D-GalN (700 mg/kg) plus 15 μg/kg LPS or 15 μg/kg TNF-α was injected. Treatment with thiolutin (THL, Cayman, 11350), a small molecule inhibitor of BRISC [43], was performed by two intraperitoneal injections (2.5 mg/kg) 1 h before and 1 h after the D-GalN/LPS or D-GalN/TNF-α administration. Experiments were performed blinded to the identity of mouse genotype and the different treatment groups.

### Bone marrow transplantation

Bone marrow transplantation (BMT) experiments with KCs pre-depletion were performed as previously described with slight modifications [40]. To deplete KCs and accelerate hepatic macrophage turnover with BM cells, recipient mice were intravenously injected with 200 μl clodronate liposomes (ClodronateLiposomes.org, CP-010-010) 24 h before being subjected to lethal irradiation (4 Gy + 4 Gy, 1 h apart). We then injected 1 × 10^7^ BM cells of donor mice into the tail veins of the recipient mice within 6 h after irradiation. To examine the relative importance of ABRO1 in hematopoietic cells, BM cells from WT mice (CD45.2) or *Abro1*^−/−^ mice (CD45.2) were transplanted into WT mice (CD45.1). Conversely, BM cells from WT mice (CD45.1) were transplanted into WT mice (CD45.2) or *Abro1*^−/−^ mice (CD45.2) to examine the role of ABRO1 in non-hematopoietic cells. Control groups were generated by transferring BM cells from WT mice (CD45.2) to WT mice (CD45.2) and from *Abro1*^−/−^ mice (CD45.2) to *Abro1*^−/−^ mice (CD45.2). Engraftment was confirmed by flow cytometry in the peripheral blood and livers at 8 weeks after transplantation. 10 weeks after BMT, mice were subjected to D-GalN/LPS treatment.

### Cell isolation

Primary hepatocytes and KCs were isolated as described previously with slight modifications [55]. In situ liver perfusion was performed at 7 ml/min via the portal vein for 6 min with pre-perfusion buffer (137 mM NaCl, 5.4 m M KCl, 0.8 m M Na_2_HPO_4_·12H_2_O, 0.6 mM NaH_2_PO_4_·2H_2_O, 10 mM HEPES, 0.5 mM EGTA, 4.2 mM NaHCO_3_, 5 mM glucose, pH 7.4) at 37 °C until the liver was completely discolored and 5 ml/min for 4 min with perfusion buffer [Dulbecco’s modified Eagle’s medium (DMEM, Cell Technology, M1805), 1% BSA (Amresco, 0332), 0.08% collagenase IV (Gibco, 17104019), 0.008% Trypsin inhibitor (Sigma-Aldrich, T9128), 5 mM CaCl_2_, pH 7.4]. After the two-step collagenase perfusion, the liver tissue was dissected, finely smashed by forceps in a sterile petri dish containing H-DMEM, 1% BSA, 5 mM MgCl_2_, 0.01% DNase I (Applichem, A3778), and passed through a 70 μm cell strainer. The single-cell suspension was centrifuged at 50 × *g* for 5 min at 4 °C. The pellet was resuspended in 10 ml of 50% Percoll (GE, 17089102) and centrifuged at 50 × *g* for 10 min at 4 °C without the brake. The hepatocyte pellet was collected and washed twice with PBS. For KCs isolation, the supernatant was collected by high-speed centrifugation at 600 × *g* for 10 min at 4 °C. The pellet was resuspended in 3 ml of 24% OptiPrep (Axis-Shield, AS1114542), and then 3 ml of 17.6% Optiprep, 3 ml of 8.4% Optiprep, and 2 ml of DMEM was carefully loaded in turn. Centrifuge at 1420 *g* for 20 min at 4 °C without the brake. The cell fraction between the interface of the 8.4% and 17.6% Optiprep was carefully transferred to a clean collection tube and washed with ice-cold sorting buffer (137 mM NaCl, 5.4 mM KCl, 0.8 mM Na_2_HPO_4_·12H_2_O, 0.6 mM NaH_2_PO_4_·2H_2_O, 25 mM HEPES, 5 mM EDTA, 4.2 mM NaHCO_3_, 5 mM glucose, 0.2% BSA, pH 7.4). Enriched KCs were pelleted by centrifugation at 600 × *g* for 10 min at 4 °C and further purified by MACS using respective surface markers F4/80 as per manufacturer’s instructions (Miltenyi Biotec, 130-110-443). KCs were determined by flow cytometry and cultured in DMEM supplemented with 20% heated inactivated FBS and 20 ng/ml M-CSF (Peprotech, 315-02) at 37 °C and 5% CO_2_.

Peritoneal resident macrophages were isolated from peritoneal cavities by lavaged with 5 ml of ice-cold RPMI-1640 medium (Gibco, 31800-022) containing 5 mM EDTA. The peritoneal fluid was centrifuged at 800 × *g* for 5 min at 4 °C and washed with ice-cold RPMI-1640 medium. The cell pellet was resuspended in warm RPMI-1640 medium and allowed to adhere for 2 h at 37 °C and 5% CO_2_. Non-adherent cells were removed by washing with warm RPMI-1640 medium for 3 times. The peritoneal resident macrophages were cultured in RPMI-1640 medium supplemented with 10% heated inactivated FBS and 1% P/S with 5% CO_2_ at 37 °C. The purification was determined by flow cytometry.

For bone marrow neutrophil isolation, femurs and tibias were removed and flushed with normal saline (NS), followed by centrifugation at 800 × *g* for 5 min. The cell pellet was resuspended in 10 ml of 0.2% NS. After lysing for 20 s, restore the osmolarity with 10 ml 1.6% NaCl. Pour the suspension into another 50 ml conical tube through a 40 μm cell strainer. Pellet the suspension at 400 × *g* for 5 min, resuspend in 5 ml NS, and carefully layer it over 5 ml 66% Percoll (in 1× final NS). Centrifuge at 25 °C for 30 min at 1000 × *g* without brake, and mature neutrophils were at the end of the gradient-centrifugation. Transfer the pellet to another 15 ml tube, and wash twice with NS. Centrifuge at 800 × *g* for 30 min, resuspend the pellet in RPMI-1640 medium complemented with 1% FBS, 1% P/S.

BMDMs were obtained as described previously [23]. Briefly, BM cells were collected and cultured in RPMI-1640 medium complemented with 10% heated inactivated FBS, 1% P/S, and 20–30% L929-conditioned media to differentiate into BMDMs. On day 7, all adherent cells became mature macrophages. L929 cells were cultured in RPMI-1640 medium complemented with 10% FBS and 1% P/S.

### Detection of NF-κB activation

The MSCV fragment of pCDH-MSCV-MCS-EF1-copGFP-T2A-Puro expression vector (System Biosciences, CD713B-1) was replaced by the NF-κB-RE-minP-luc2P fragment of pGL4.32 [luc2P/NF-κB-RE/Hygro] Vector (Promega, E849A) to generate the lentiviral NF-κB-luciferase reporter construct. KCs were incubated with the lentivirus at a multiplicity of infection (MOI) of 10 in the culture medium containing 4 μg/ml polybrene (Sigma, H9268) for 12 h. After another 48 h of culture in regular medium, cells were stimulated with LPS for 3 h and lysed in Passive Lysis Buffer (Promega, E194A). The luminescence levels were measured using the Dual-Luciferase Reporter Assay System (Promega, E1910) according to the manufacturer’s instructions. Nuclear and cytoplasmic proteins of KCs treated with or without LPS were extracted by NE-PER™ Nuclear and Cytoplasmic Extraction Reagents (Thermo Fisher Scientific, 78833). Protein concentration was quantified using a NanoDrop 2000c spectrophotometer (Thermo Fisher Scientific). The DNA binding activity of NF-κB was analyzed by the NF-κB p65 Transcription Factor Assay Kit (Abcam, ab133112), and NF-κB nuclear translocation was determined by western blot. For the rescue experiment, two small molecular activators of NF-κB (NF-κΒ activator 1, denoted Compounds 32, MedChemexpress, HY-134476; NF-κΒ activator 2, denoted Compounds 61, MedChemexpress, HY-134477) were solubilized in DMSO at a concentration of 5 mM. KCs were pre-treated with NF-κΒ activator 1 or NF-κΒ activator 2 for 6 h before exposed to LPS.

### Western blot

For western blot, cells were directly lysed in 2 × Laemmli sample buffer and separated by SDS-PAGE. Proteins on the gel were transferred to a PVDF membrane and then probed with indicated primary antibodies. Immune complexes on the membrane were detected with HRP-conjugated secondary antibodies and enhanced chemiluminescence reagents (Thermo Fisher Scientific, 34580). The antibodies used in this paper are listed in Supplementary Table 1. Full and uncropped Western blots are presented in Supplementary File.

### Cell cytotoxicity assay

Primary KCs were seeded in 96-well plates and stimulated as described in the figure legends. LDH release was measured by CytoTox 96 Non-Radioactive Cytotoxicity Assay (Promega, G1780). Cell proliferation was performed with CellTiter 96^®^ AQueous One Solution Cell Proliferation Assay according to the manufacturer’s instruction (Promega, G3580).

### Histological analysis, immunohistochemistry staining, and TUNEL assay

Liver tissues were excised and fixed in 4% paraformaldehyde and then embedded in paraffin. Sections were stained with hematoxylin and eosin (H&E) for morphological analysis. The necrosis was expressed as a percentage of necrotic areas of ×200 magnification per slide. The immune cell infiltration in mice livers was determined by immunohistochemistry staining with anti-mouse F4/80 (Abcam, ab111101), anti-mouse CD3 (Abcam, ab237721), or anti-mouse Ly6G (Abcam, ab25377) antibody. Immunohistochemistry staining with anti-cleaved caspase-3 antibody (Abcam, ab2302) was used to determine the activity of caspase-3. The terminal deoxynucleotidyl transferase-mediated dUTP nick end labeling (TUNEL) assay was carried out with a commercial kit (Roche, 11684817910) following the manufacturer’s recommended protocol. Nuclei were counterstained with hematoxylin. Positive cells or areas were analyzed from randomly selected 3 fields of ×200 or ×400 magnification for each sample. All images of the liver sections were captured using Nikon Digital Sight DS-U3 camera. Image analysis procedures were performed with Image Pro Plus v6.0 (Media Cybernetics, Inc).

### Detection of cytokines and serum aminotransferase

To detect the cytokines in serum, liver homogenates, and culture supernatants, Cytometric bead array (CBA) Mouse TNF Flex Set (BD Biosciences, 558299), Mouse IL-6 Flex Set (BD Biosciences, 558301), Mouse MCP-1 Flex Set (BD Biosciences, 558342), Mouse MIP-1α Flex Set (BD Biosciences, 558449), Mouse MIP-1β Flex Set (BD Biosciences, 558343) and Mouse IL-1β Flex Set (BD Biosciences, 560232) were used according to manufacturer’s instruction. Liver total protein quantification was performed with the Pierce™ BCA Protein Assay Kit (Thermo Fisher Scientific, 23227). Serum ALT and AST were measured according to the IFCC primary reference procedures at Beijing CIC Clinical Laboratory (Beijing, China).

### Flow cytometry

Peripheral blood was collected into heparin-containing tubes. Mouse BM cells were harvested from femurs and tibias, dissociated by gently passing through a 21G needle. RBCs were lysed with ammonium chloride (TIANGEN Biotech, RT122). For LPS/DGalN-induced stress hematopoiesis analysis, blood was stained with the antibody panel: anti-mouse CD45.2-PE, anti-mouse Ly6G-FITC, anti-mouse CD3e-APC, anti-mouse B220-PE-Cy7, and anti-mouse CD11b-eFluor 450. BM cells were stained with anti-mouse CD45.2-PE, anti-mouse Ly6G-APC, and anti-mouse CD11b-eFluor 450. For the engraftment analysis, blood was stained with anti-mouse CD45.1-FITC, anti-mouse CD45.2-PE, anti-mouse CD3e-APC, anti-mouse CD19-PE-Cy7, and anti-mouse CD11b-eFluor 450. Red blood cells (RBCs) were then lysed using RBC Lysis/Fixation Solution (Biolegend, 422401).

Murine liver leukocytes were isolated with a modification method [56]. In brief, after the two-step collagenase perfusion in situ, the liver tissue was dissected, finely smashed by forceps, then poured through a 40 μm strainer and centrifugated at 50 × *g* for 5 min. Supernatant was collected by centrifugation at 320 × *g* for 5 min. Resuspend the pellet in 10 ml of 30% Percoll, then centrifugate at 800 *×* *g* for 15 min without brake. Cell pellet was washed and resuspended by 2 ml ammonium chloride lysis buffer for 10 min. Cells were pelleted by centrifugation at 500 × *g* for 5 min. For liver macrophages analysis, liver leukocytes were stained with anti-mouse F4/80-FITC, anti-mouse CD45.2-PE, anti-mouse Ly6C-PE-Cy7, anti-mouse Siglec F-eFluor 660, anti-mouse Ly6G-eFluor 450, and anti-mouse CD11b-BV605. In the case of analysis of common KCs’ markers and M1/M2 polarization markers expression, liver leukocytes were further stained with anti-mouse Clec4F-AF647, anti-mouse Tim4-AF647, anti-mouse CD80-APC, anti-mouse CD86-APC, anti-mouse CD163-APC, or anti-mouse CD206-APC. To stain intracellular cytokines, liver leukocytes were first stained for the cell surface antigens by incubation with anti-mouse F4/80-FITC, anti-mouse CD45.2-PE, anti-mouse Ly6C-PE-Cy7, anti-mouse Siglec F-BV421, anti-mouse Ly6G-eFluor 450, and anti-mouse CD11b-BV605 for 30 min. Cells were then fixed and permeabilized with Fixation Buffer (Biolegend, 420801) and Intracellular Staining Perm Wash Buffer (Biolegend, 421002) prior to the addition of intracellular stains for anti-mouse TNFα-APC or anti-mouse MCP1-APC. The fluorochrome-labeled antibodies used in this paper are listed in Supplementary Table 1.

The number of leukocytes was counted by 123-count eBeads Counting Beads (eBioscience, 01-1234). Dead cells were excluded with Fixable Viability Dye eFluor 780 (eBioscience, 65-0865-14) if necessary. Flow cytometric analysis was performed using a BD LSRFortessa (BD Biosciences) and at least 10,000 target cells were acquired.

### RNA extraction and real-time PCR

Total RNA was extracted by TRIzol (Thermo Fisher Scientific, 15596026) and reverse-transcribed using RevertAid First Strand cDNA Synthesis Kit (Thermo Fisher Scientific, K1622). The cDNAs were amplified with SYBR Green Realtime PCR Master Mix (TOYOBO, QPK-201) by LightCycler 480 real-time PCR detection system (Roche). Relative gene expression was evaluated by the ΔCT method, and *Gapdh* was used as an internal control. Genes-specific primers were designed by Primer Bank. The primers used in this paper are listed in Supplementary Table 2.

### Clinical datasets analysis

For published clinical datasets analysis, datasets were downloaded from Gene Expression Omnibus (GEO) repository (GSE38941) and processed using oligo package. Raw data were normalized by the robust multiarray average (RMA) method [57].

### Statistical analyses

Statistics were calculated with GraphPad Prism 7 (GraphPad Software). All experiments were conducted using 3–13 mice or repeated three independent times with cells. The distribution of variables is tested by the Kolmogorov-Smirnov test. Statistical analysis was carried out using a standard two-tailed unpaired Student’s *t*-test for single comparisons, one- or two-way analysis of variance (ANOVA) for multiple comparisons, and a log-rank (Mantel-Cox) test for survival analysis. Results were expressed as means ± standard error of the mean (SEM). Expression scores were shown as box plots, with the horizontal lines representing the median; the bottoms and tops of the boxes represent the 25th and 75th percentiles, respectively, and the vertical bars represent the range of data. *P* value < 0.05 was considered statistically significant.

### Reporting summary

Further information on research design is available in the Nature Research Reporting Summary linked to this article.

### Supplementary information

Supplementary figure legends

Supplementary table 1

Supplementary table 2

Supplementary Figure 1

Supplementary Figure 2

Supplementary Figure 3

Supplementary Figure 4

Supplementary Figure 5

Supplementary Figure 6

Supplementary Figure 7

Supplementary Figure 8

Supplementary Figure 9

Original Data File

Reporting Summary

## Data Availability

All data are available from the corresponding author upon reasonable request. Original western blots images are available in Supplementary Materials.
