# Supplementary material for: BRISC is required for optimal activation of NF-κB in Kupffer cells induced by LPS and contributes to acute liver injury
Source: Cell Death Dis. 2023 Nov 15;14(11):743. doi: 10.1038/s41419-023-06268-z (PMC10651896; doi:10.1038/s41419-023-06268-z)
Supplement: Supplementary file 1 — Supplementary figure legends [file 41419_2023_6268_MOESM1_ESM.docx]

**Supplementary Figure Legends**

**Fig. S1 The purity of the isolated KCs and hepatocytes. A** Flow cytometry gating strategy for KCs. KCs are defined as Clec4F^+^. **B** KCs after 12 h of culture (100×). **C** Hepatocytes after 4 h of culture (100× and 200×).

**Fig. S2 D-GalN/LPS-induced hepatic inflammation is attenuated in BRISC-deficient mice. A** Immunohistochemistry (IHC) staining analysis of CD3^+^ cells of liver sections from WT and *Abro1*^−/−^ mice treated with D-GalN/LPS for the indicated times (N=3-4). Positive cells per high-power field (× 400) were counted. WT and *Abro1*^−/−^ mice were treated with D-GalN/LPS for the indicated times (N=4-6). **B** Flow cytometry analysis of the percentage of CD3^+^ (T lymphocytes), B220^+^ (B lymphocytes), CD11b^+^ (myelocytes), and CD11b^+^Ly6G^+^ (Neutrophils) cells in peripheral blood. **C** The percentage and the number of CD11b^+^ and CD11b^+^Ly6G^+^ cells in BM. **D** CBA analysis of the serum levels of TNF-α, IL-6, and MCP-1 in WT and *Brcc3*^−/−^ mice treated with D-GalN/LPS or PBS for 6 h (N=3-5). Scale bar, 50 μm. Data are presented as mean ± SEM; **P* < 0.05, ***P* <0.01; two-tailed unpaired *t*-test.

**Fig. S3 BRISC deficiency selectively impairs LPS-induced production of proinflammatory cytokines in KCs *in vivo*. A** Flow cytometry gating strategy for liver macrophages. Plots were first gated on single live CD45^+^Ly6G^-^SiglecF^-^ cells. KCs are further defined as F4/80^hi^CD11b^lo^, inflammatory Mos as F4/80^int^CD11b^int^Ly6C^hi^, and MoMs as F4/80^int^CD11b^int^Ly6C^lo^. **B** The percentage of TNF-α- and MCP-1-producing Mos, MoMs, and KCs in WT and *Abro1*^−/−^ mice treated with D-GalN/LPS or PBS for 1 h (N=4-6). **C** The percentage of TNF-α- and MCP-1-producing Mos, MoMs, and KCs in WT and *Brcc3*^−/−^ mice treated with D-GalN/LPS for 1 h (N=3-6). Data are presented as mean ± SEM; **P* < 0.05, ***P* <0.01, ****P* <0.001; two-tailed unpaired *t*-test.

**Fig. S4 ABRO1 deficiency has no effect on hematopoietic reconstitution and liver macrophages replacement after bone marrow transplantation.** BM chimeras were generated by BMT with depletion of KCs prior to irradiation (N=3-7). **A, C** BM cells from WT mice (CD45.2) or *Abro1*^−/−^ mice (CD45.2) were transplanted into WT mice (CD45.1). **B, D** BM cells from WT mice (CD45.1) were transplanted into WT mice (CD45.2) or *Abro1*^−/−^ mice (CD45.2). **A, B** Engraftment in the peripheral blood and the reconstitution of CD3^+^, CD19^+^, and CD11b^+^ cells were confirmed by flow cytometry at 8 weeks after transplantation. **C, D** Flow cytometry analysis of the percentage of donor-derived Mos, MoMs, and KCs in the livers of recipient mice. Data are presented as mean ± SEM; two-tailed unpaired *t*-test.

**Fig. S5 BRISC has a limited role in TNF-α-induced signaling pathway activation in hepatocytes. A** WT and *Abro1*^−/−^ primary hepatocytes were treated with 1 mg/ml D-GalN and 50 ng/ml TNF-α for the indicated times. The cell cytotoxicity and proliferation activity were determined by lactate dehydrogenase (LDH) assay and MTS assay respectively. **B** WT and *Abro1*^−/−^ hepatocytes were stimulated with 50 ng/ml TNF-α for various times. Immunoblot analysis of the indicated target proteins. Data are presented as mean ± SEM; **P* < 0.05, ***P* <0.01, ****P* <0.001; two-tailed unpaired *t*-test.

**Fig. S6 Construction strategy and knockout efficiency of ABRO1 in cell-specific ABRO1-deficient mice. A** Construction strategy of *Abro1*^flox/flox^ mice. Knockout efficiency of ABRO1 in **B** *Abro1*^flox/flox^*Alb-Cre*^+^ mice, **C** *Abro1*^flox/flox^*Lyz2-Cre*^+^ mice, and **D** *Abro1*^flox/flox^*Clec4f-Cre*^+^ mice. **E** The protein levels of BRISC complex components in WT and *Abro1*^−/−^ cells. BMDM, bone marrow-derived macrophages; NEUT, neutrophils; PM, resident peritoneal macrophages.

**Fig. S7 Hepatocyte-specific deletion of ABRO1 has no significant improvement in D-GalN/LPS-induced liver injury.** *Abro1*^flox/flox^ and *Abro1*-HKO mice were challenged with a sublethal dose of D-GalN/LPS (N=6-11). Liver injury was evaluated by **A** serum ALT level and **B** H&E staining 6 h after D-GalN/LPS injection. Necrotic area was shown as a percentage of the total field area. Scale bar, 50 μm. Data are presented as mean ± SEM; two-tailed unpaired *t*-test.

**Fig. S8 KCs development under steady-state is not affected in BRISC-deficient mice. A** The percentage and number of KCs in WT and *Abro1*^−/−^ mice (N=5). **B** Expression of common KCs’ markers of WT and *Abro1*^−/−^ KCs (N=5). **C** The mean fluorescence intensity of CD80, CD86, CD163, and CD206 on WT and *Abro1*^−/−^ KCs (N=5-6). Data are presented as mean ± SEM; two-tailed unpaired *t*-test.

**Fig. S9 BRISC inactivation did not affect the survival of KCs.** **A** MTS analysis of WT and *Abro1*^−/−^ KCs stimulated with various doses of LPS for 3 h. **B** WT KCs pre-treated with 25 nM or 50 nM THL or vehicle control for 2 h were left unstimulated or stimulated with 1 μg/ml LPS for 6 h. The cell cytotoxicity was determined by LDH assay. Data are presented as means ± SEM; **P* < 0.05, ***P* <0.01; two-way ANOVA with Dunnett’s multiple comparisons test (**A**) or two-tailed unpaired *t*-test (**B**).
