# Supplementary material for: BRISC is required for optimal activation of NF-κB in Kupffer cells induced by LPS and contributes to acute liver injury
Source: Cell Death Dis. 2023 Nov 15;14(11):743. doi: 10.1038/s41419-023-06268-z (PMC10651896; doi:10.1038/s41419-023-06268-z)
Supplement: Supplementary file 2 — Supplementary table 1 [file 41419_2023_6268_MOESM2_ESM.docx]

**Supplementary Table 1. Antibodies used in this paper**

| Antibody | Supplier | Catalog Number |
| --- | --- | --- |
| rabbit monoclonal anti-BRCC3 | Cell Signaling Technology | 18215 |
| rabbit monoclonal anti-BRE | Cell Signaling Technology | 12457 |
| rabbit monoclonal anti-MERIT40 | Cell Signaling Technology | 12711 |
| rabbit polyclonal anti-ABRO1 | ABclonal | generated by ABclonal |
| rabbit monoclonal anti-phospho-NF-κB p65 (Ser536) | Cell Signaling Technology | 3033 |
| rabbit monoclonal anti-NF-κB p65 | Cell Signaling Technology | 8242 |
| rabbit monoclonal anti-Phospho-IκBα (Ser32) | Cell Signaling Technology | 2859 |
| mouse monoclonal anti-IκBα | Cell Signaling Technology | 4814 |
| rabbit monoclonal anti-Phospho-JNK (Thr183/Tyr185) | Cell Signaling Technology | 4668 |
| rabbit polyclonal anti-JNK | Cell Signaling Technology | 9252 |
| rabbit monoclonal anti-Phospho-p38 (Thr180/Tyr182) | Cell Signaling Technology | 4511 |
| rabbit monoclonal anti-p38 | Cell Signaling Technology | 8690 |
| rabbit monoclonal anti-Phospho-p44/42 MAPK (Erk1/2)(Thr202/Tyr204) | Cell Signaling Technology | 4370 |
| rabbit monoclonal anti-p44/42 MAPK (Erk1/2) | Cell Signaling Technology | 4695 |
| mouse monoclonal anti-Lamin A/C | Cell Signaling Technology | 4777 |
| mouse monoclonal anti-β-Tubulin | ABclonal | AC021 |
| rabbit monoclonal anti-GAPDH | ABclonal | AC002 |
| goat anti-mouse IgG | ABclonal | AS003 |
| goat anti-rabbit IgG | ABclonal | AS014 |
| anti-mouse CD45.2-PE | eBioscience | 12-0454-82 |
| anti-mouse Ly6G-FITC | eBioscience | 11-9668-82 |
| anti-mouse CD3e-APC | eBioscience | 17-0031-82 |
| anti-mouse B220-PE-Cy7 | eBioscience | 25-0452-82 |
| anti-mouse CD11b-eFluor 450 | eBioscience | 48-0112-82 |
| anti-mouse Ly6G-APC | eBioscience | 11-0453-82 |
| anti-mouse CD45.1-FITC | eBioscience | 11-0453-82 |
| anti-mouse CD19-PE-Cy7 | eBioscience | 25-0193-82 |
| anti-mouse CD45.2-PE | Biolegend | 109808 |
| anti-mouse F4/80-FITC | eBioscience | 11-4801-82 |
| anti-mouse Ly6C-PE-Cy7 | eBioscience | 25-5932-82 |
| anti-mouse Siglec F-eFluor 660 | eBioscience | 50-1702-82 |
| anti-mouse Ly6G-eFluor 450 | eBioscience | 48-9668-82 |
| anti-mouse CD11b-BV605 | Biolegend | 101257 |
| anti-mouse Clec4F-AF647 | Biolegend | 156803 |
| anti-mouse Tim4-AF647 | Biolegend | 130007 |
| anti-mouse CD80-APC | Biolegend | 104713 |
| anti-mouse CD86-APC | Biolegend | 105011 |
| anti-mouse CD163-APC | Biolegend | 155305 |
| anti-mouse CD206-APC | Biolegend | 141707 |
| anti-mouse Siglec F-BV421 | Biolegend | 155509 |
| anti-mouse TNFα-APC | Biolegend | 506308 |
| anti-mouse MCP1-APC | Biolegend | 505909 |
