# Supplementary material for: BRISC is required for optimal activation of NF-κB in Kupffer cells induced by LPS and contributes to acute liver injury
Source: Cell Death Dis. 2023 Nov 15;14(11):743. doi: 10.1038/s41419-023-06268-z (PMC10651896; doi:10.1038/s41419-023-06268-z)
Supplement: Supplementary file 3 — Supplementary table 2 [file 41419_2023_6268_MOESM3_ESM.docx]

**Supplementary Table 2. Primers used in this paper**

| Target | Type | Primer sense | Sequence (5’ → 3’) |
| --- | --- | --- | --- |
| *mIl1b* | RT-PCR | F | GAAATGCCACCTTTTGACAGTG |
|  |  | R | TGGATGCTCTCATCAGGACAG |
| *mTnfa* | RT-PCR | F | CTGAACTTCGGGGTGATCGG |
|  |  | R | GGCTTGTCACTCGAATTTTGAGA |
| *mIl6* | RT-PCR | F | CTGCAAGAGACTTCCATCCAG |
|  |  | R | AGTGGTATAGACAGGTCTGTTGG |
| *mMcp-1* | RT-PCR | F | TTAAAAACCTGGATCGGAACCAA |
|  |  | R | GCATTAGCTTCAGATTTACGGGT |
| *mGapdh* | RT-PCR | F | AGGTCGGTGTGAACGGATTTG |
|  |  | R | GGGGTCGTTGATGGCAACA |
