# Supplementary figures and images for: BRISC is required for optimal activation of NF-κB in Kupffer cells induced by LPS and contributes to acute liver injury
Source: Cell Death Dis. 2023 Nov 15;14(11):743. doi: 10.1038/s41419-023-06268-z (PMC10651896; doi:10.1038/s41419-023-06268-z)

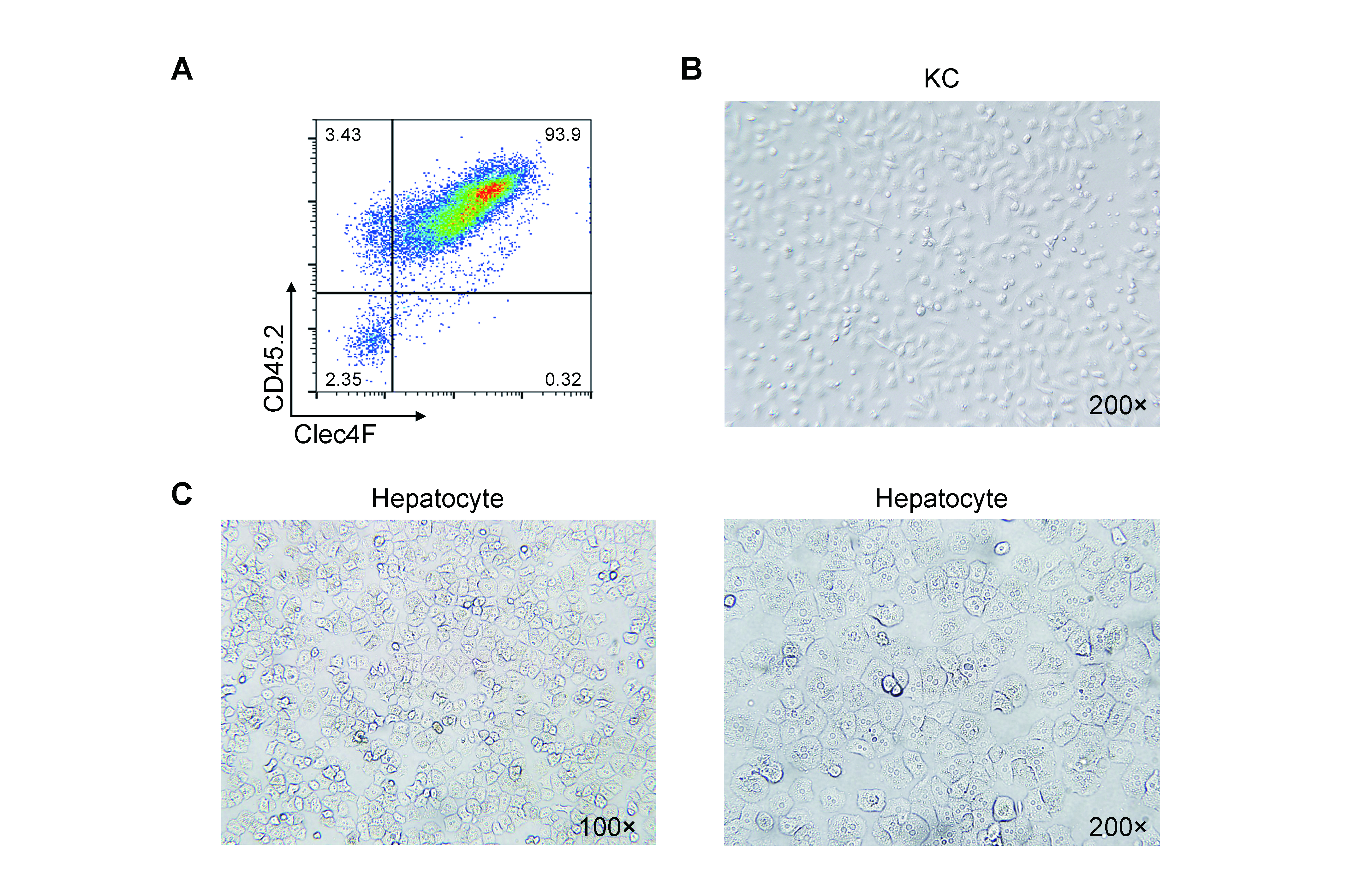

Supplement: Supplementary file 4 — Supplementary Figure 1 [file 41419_2023_6268_MOESM4_ESM.tif]

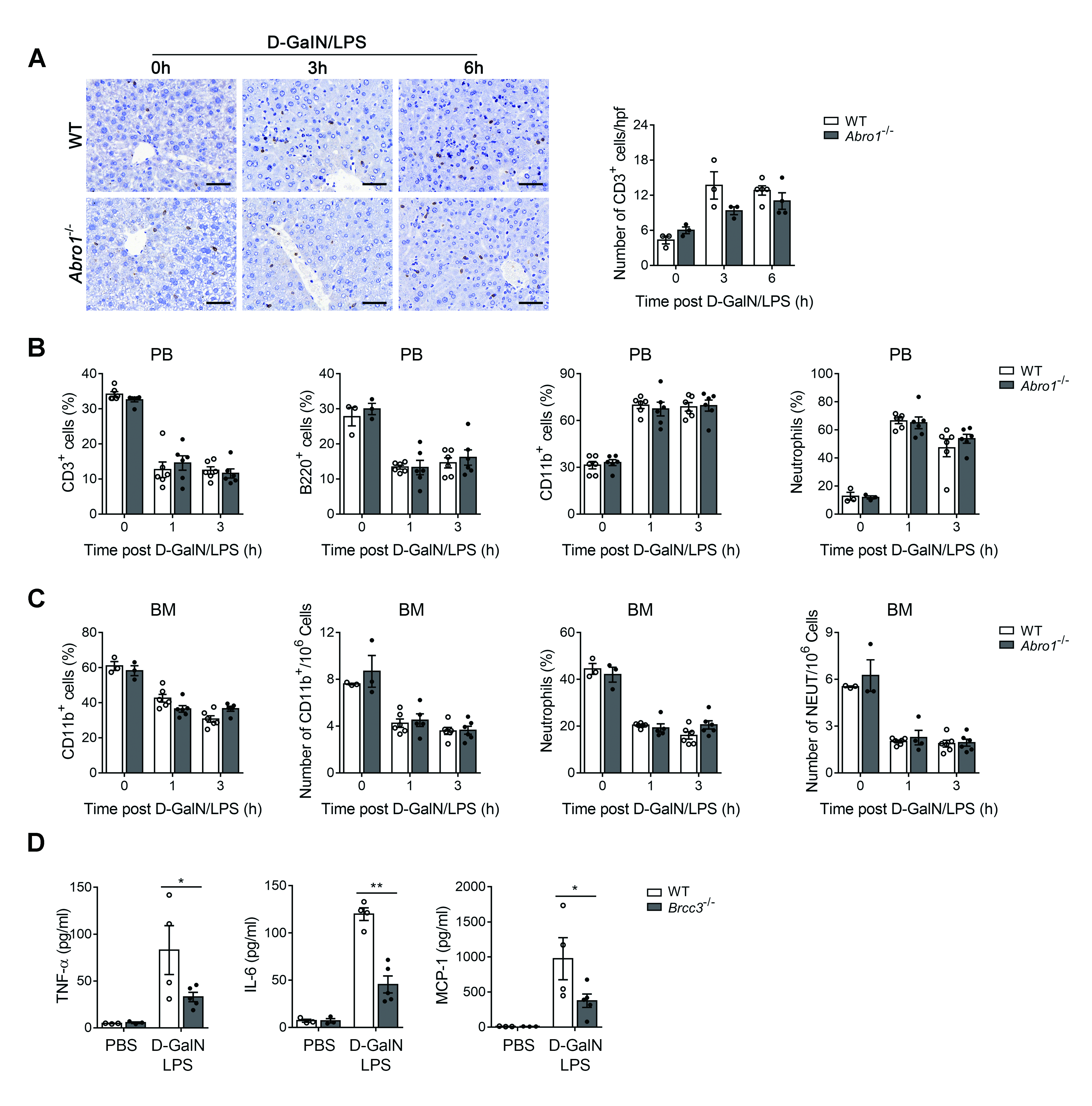

Supplement: Supplementary file 5 — Supplementary Figure 2 [file 41419_2023_6268_MOESM5_ESM.tif]

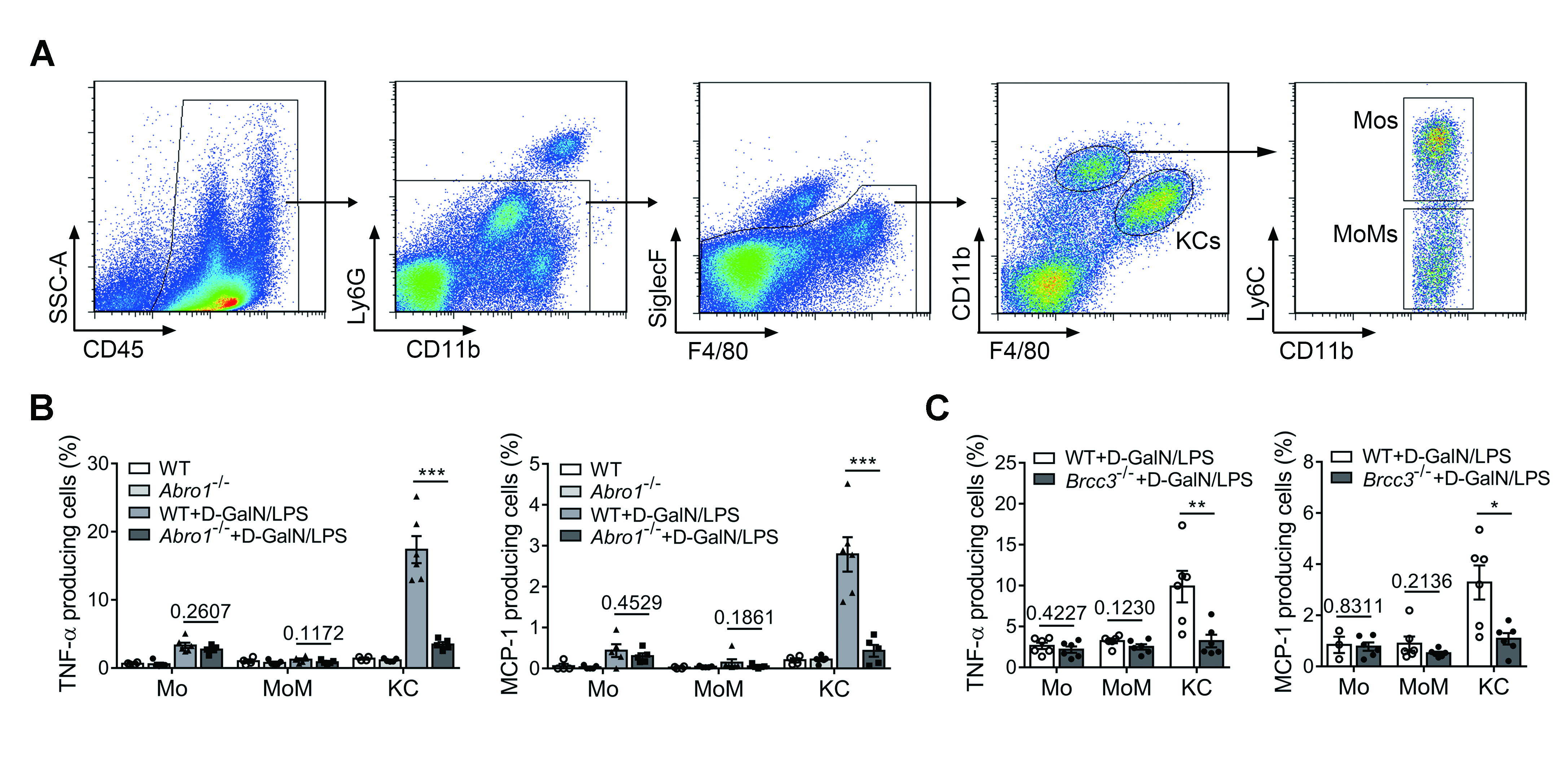

Supplement: Supplementary file 6 — Supplementary Figure 3 [file 41419_2023_6268_MOESM6_ESM.tif]

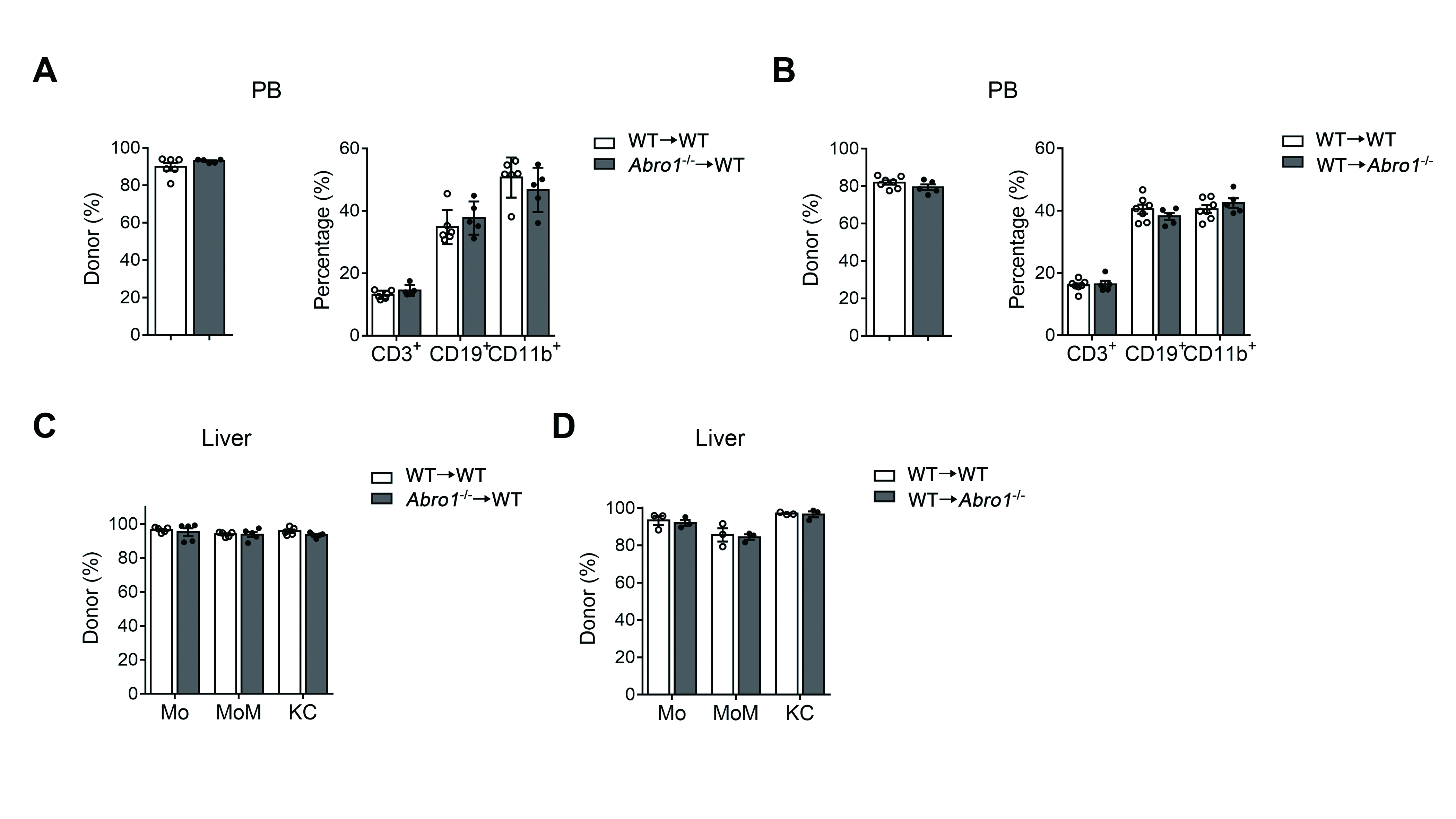

Supplement: Supplementary file 7 — Supplementary Figure 4 [file 41419_2023_6268_MOESM7_ESM.tif]

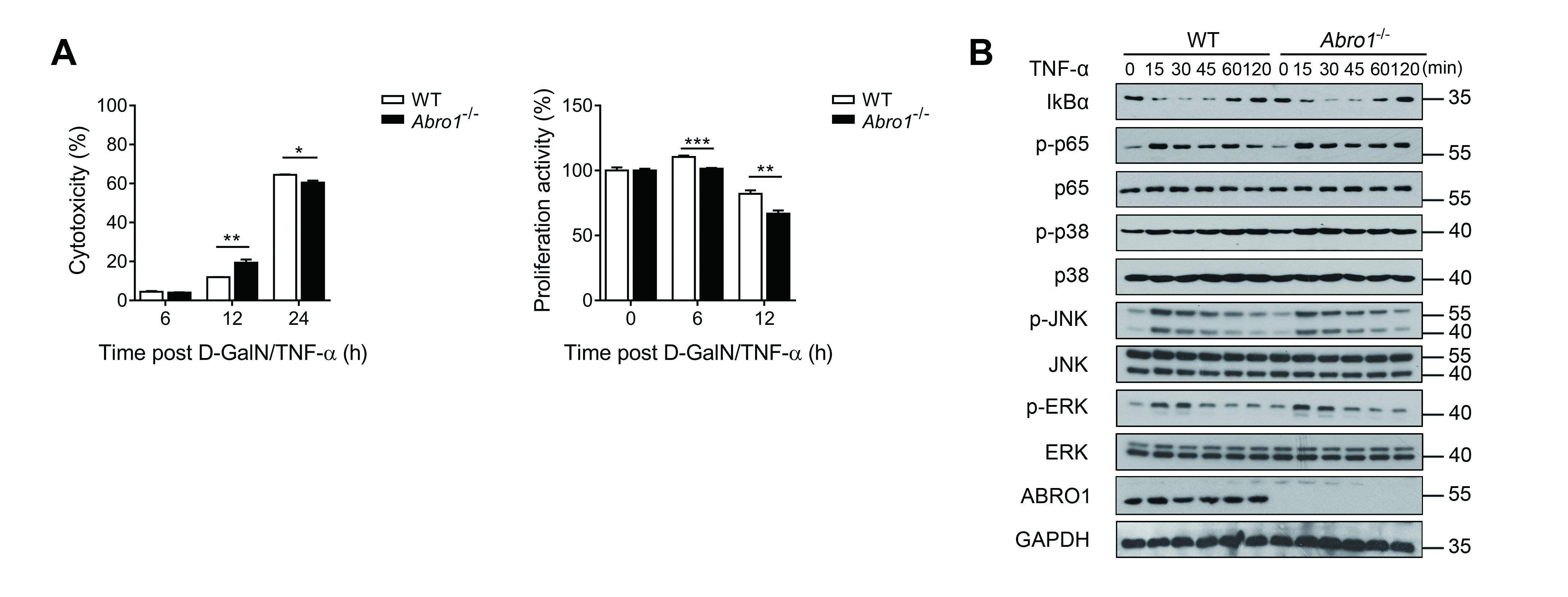

Supplement: Supplementary file 8 — Supplementary Figure 5 [file 41419_2023_6268_MOESM8_ESM.tif]

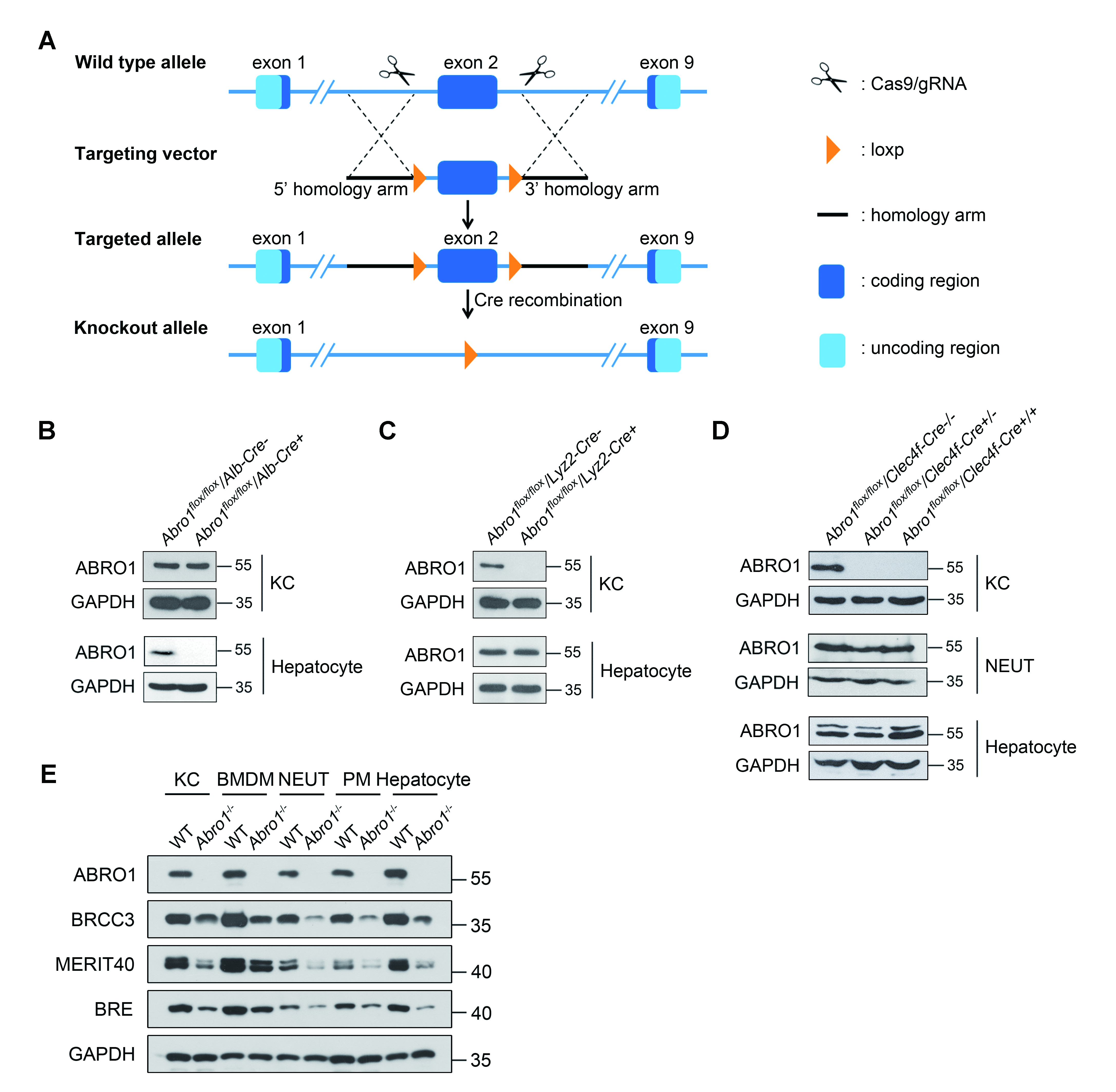

Supplement: Supplementary file 9 — Supplementary Figure 6 [file 41419_2023_6268_MOESM9_ESM.tif]

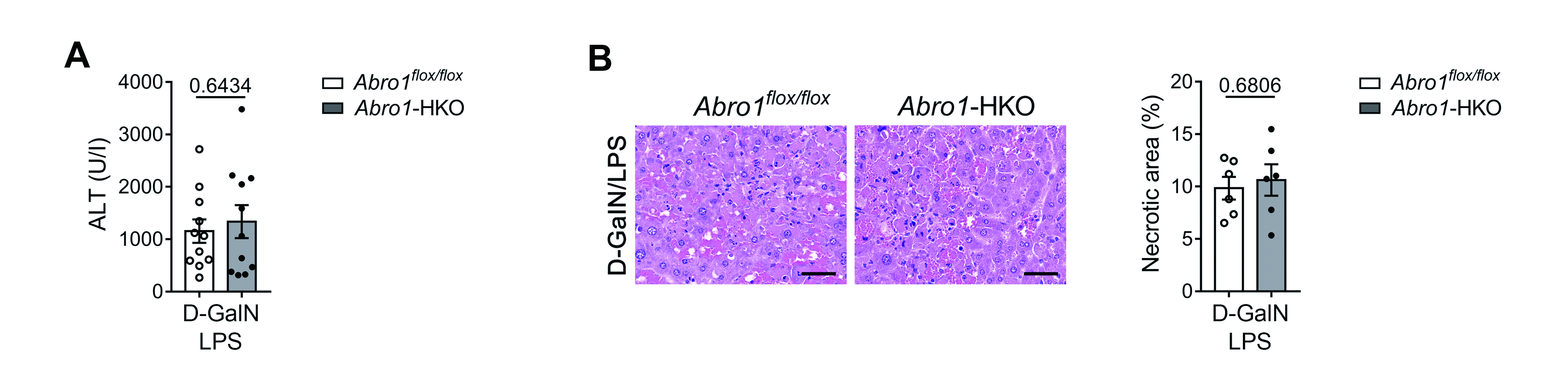

Supplement: Supplementary file 10 — Supplementary Figure 7 [file 41419_2023_6268_MOESM10_ESM.tif]

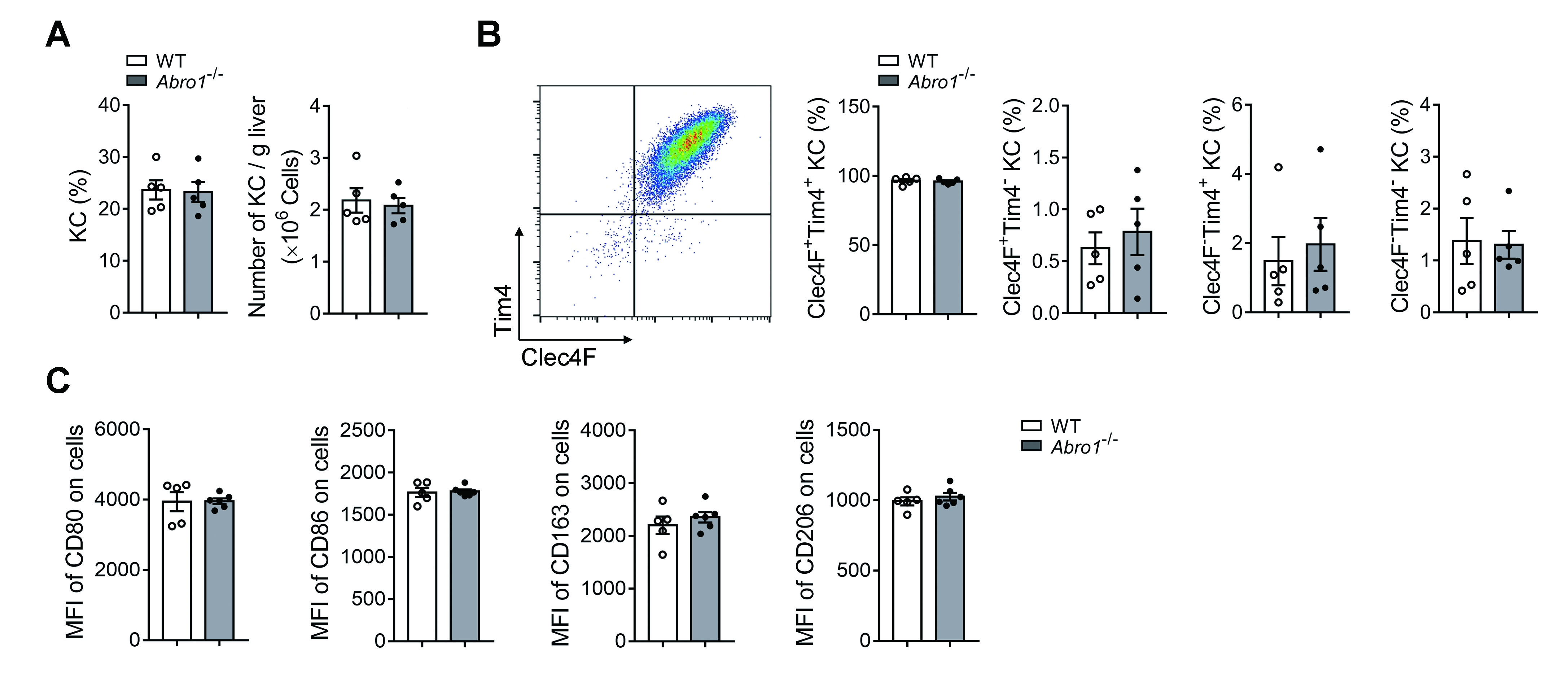

Supplement: Supplementary file 11 — Supplementary Figure 8 [file 41419_2023_6268_MOESM11_ESM.tif]

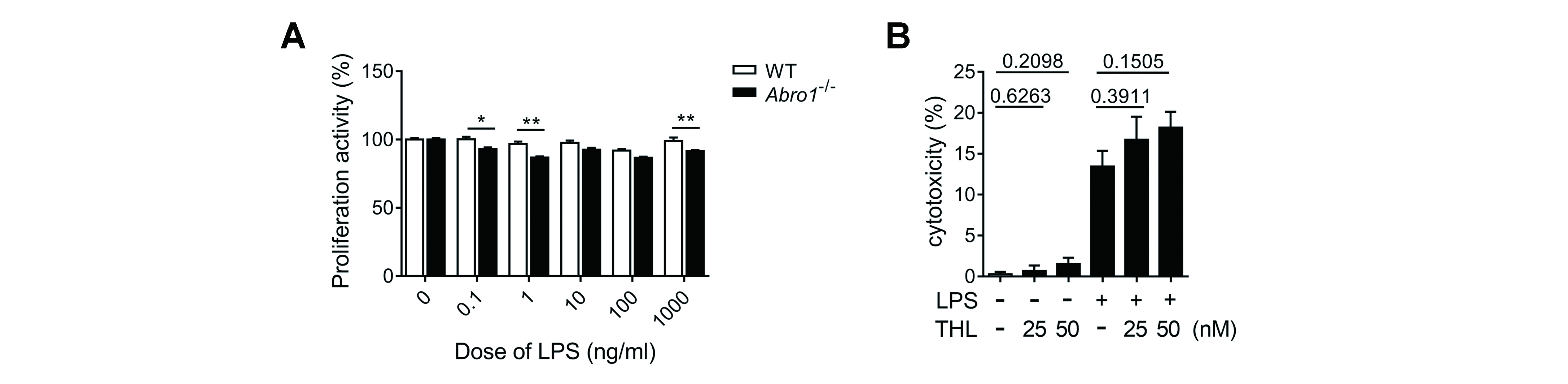

Supplement: Supplementary file 12 — Supplementary Figure 9 [file 41419_2023_6268_MOESM12_ESM.tif]
