# Supplementary material for: BRISC is required for optimal activation of NF-κB in Kupffer cells induced by LPS and contributes to acute liver injury
Source: Cell Death Dis. 2023 Nov 15;14(11):743. doi: 10.1038/s41419-023-06268-z (PMC10651896; doi:10.1038/s41419-023-06268-z)

Uncropped images for Fig.1A

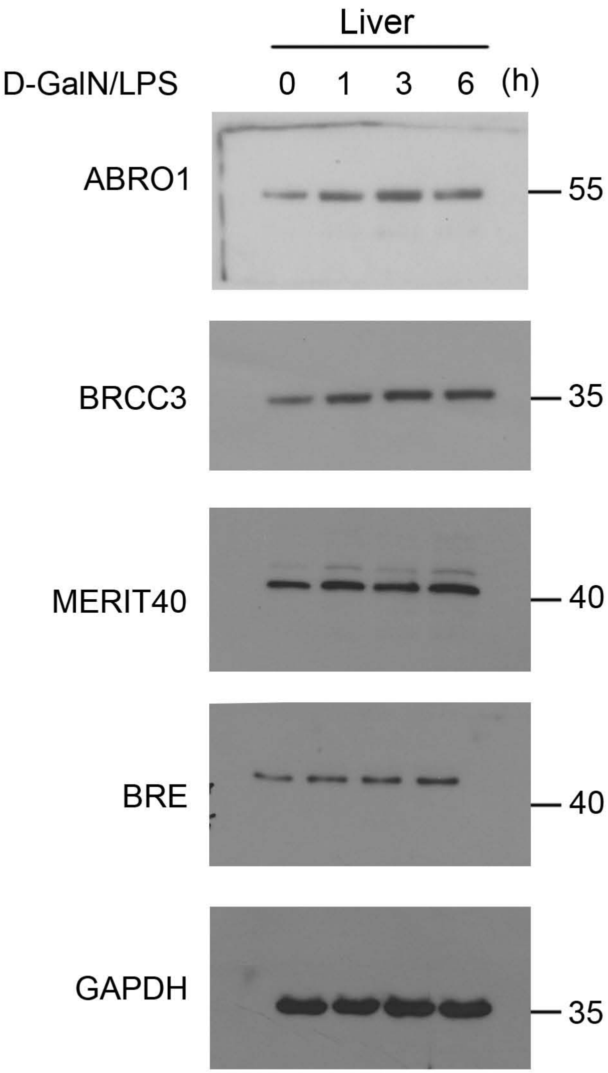

Uncropped images for Fig.1B 1C

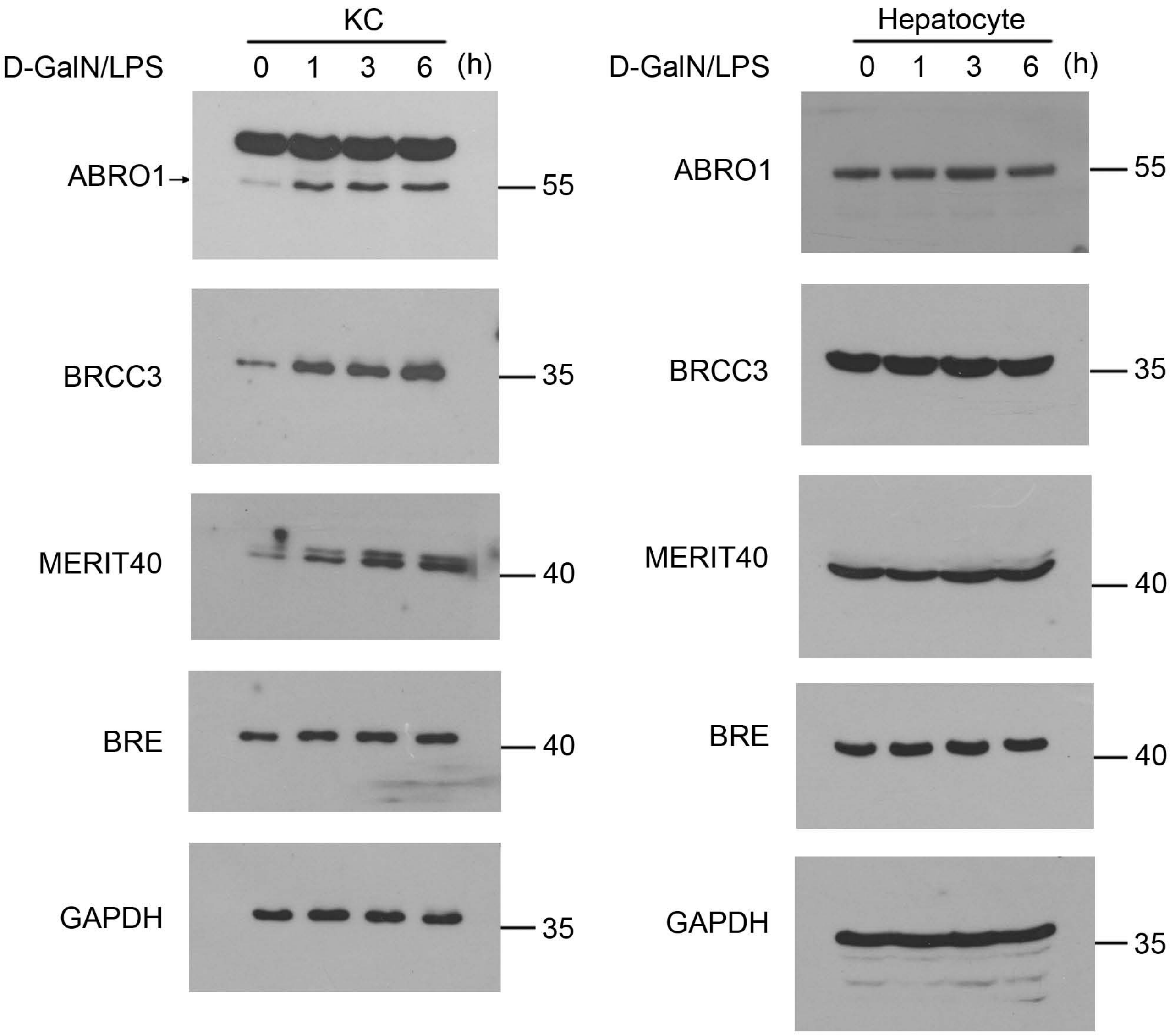

Uncropped images for Fig.1E

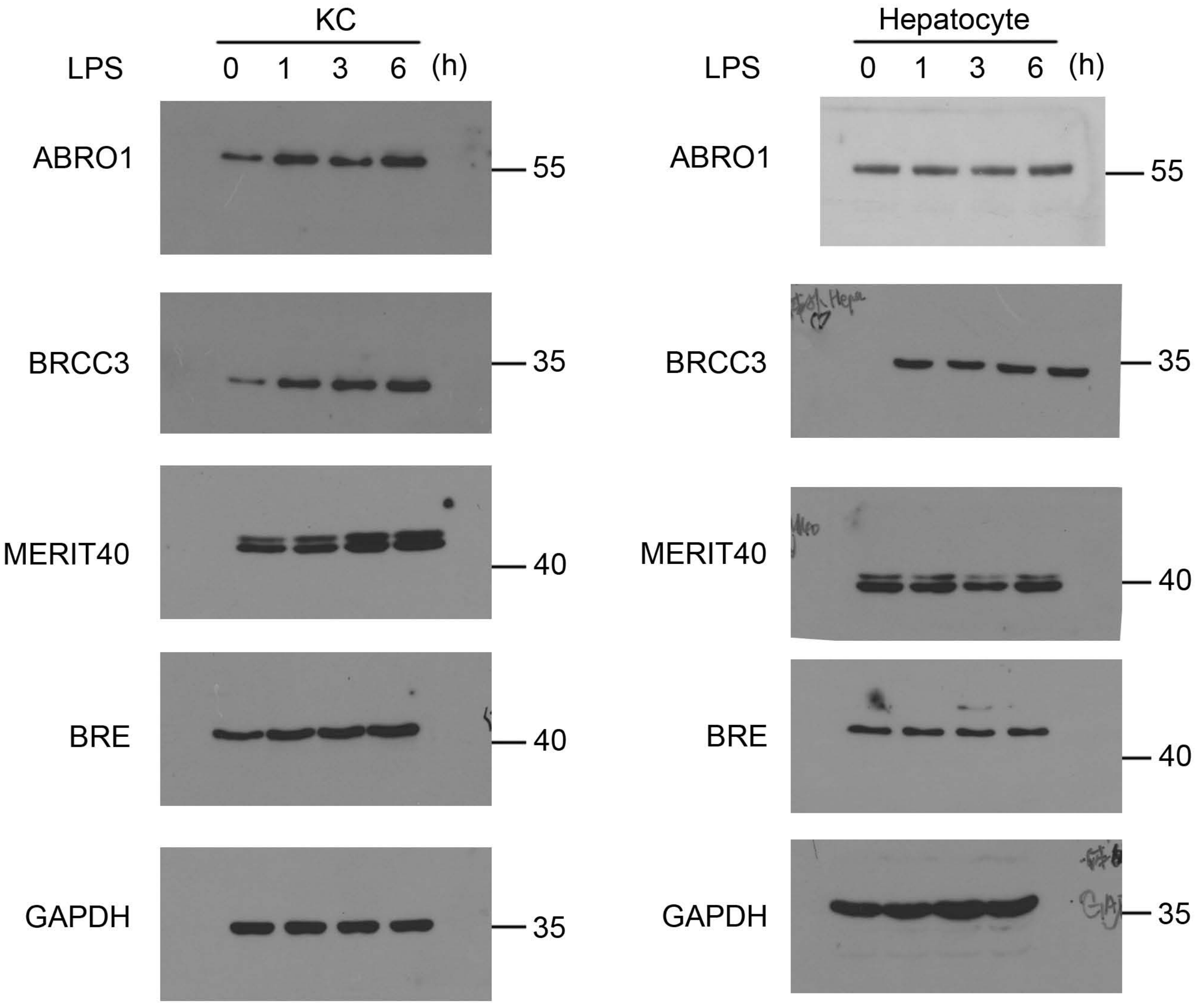

Uncropped images for Fig.7A

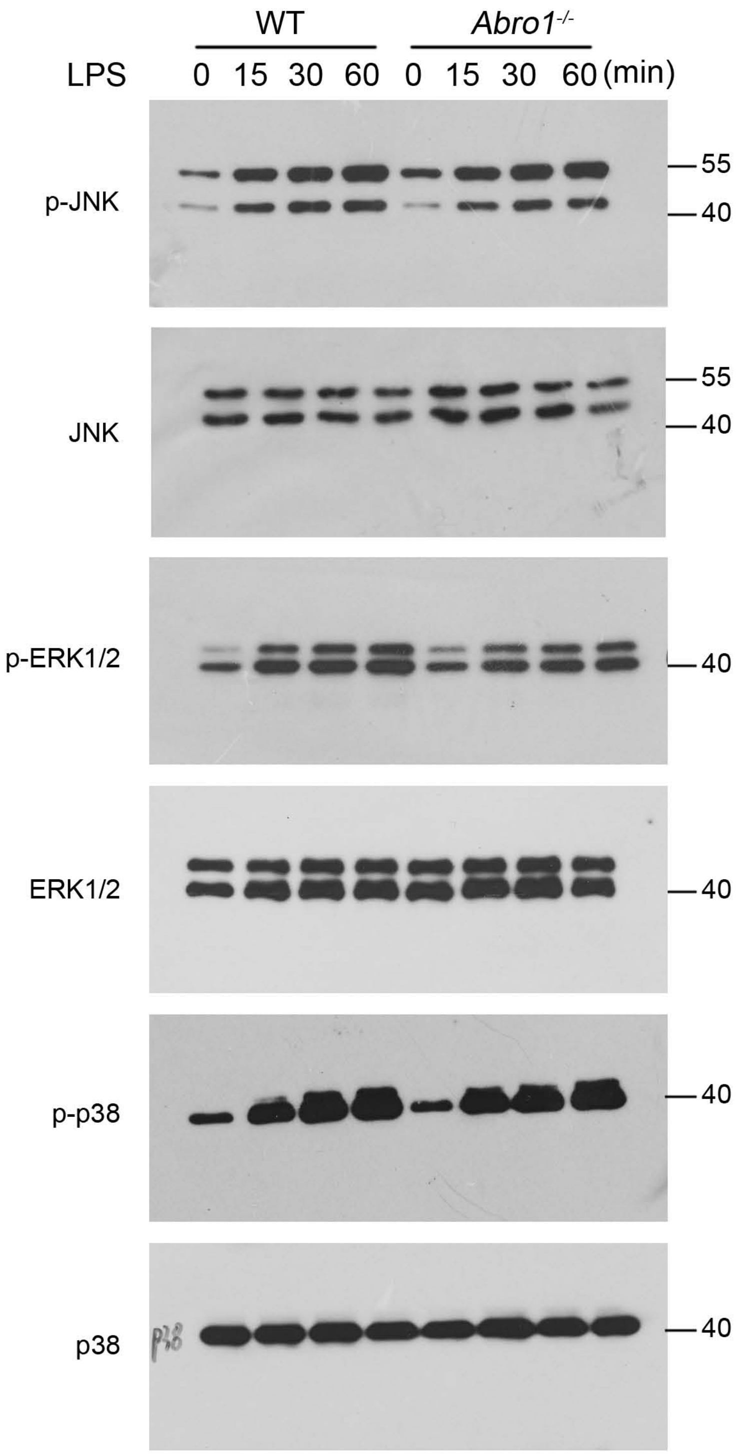

Uncropped images for Fig.7A

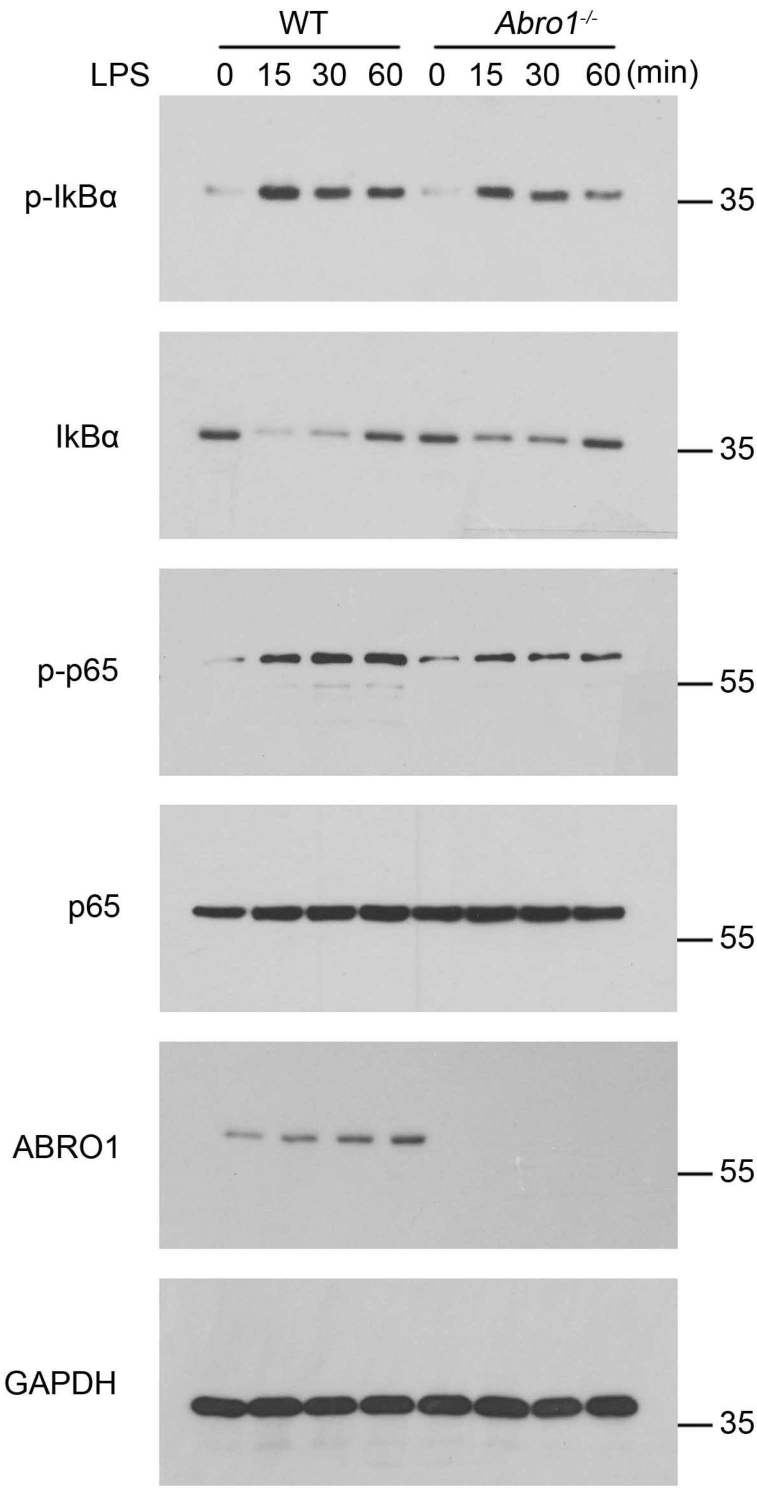

Uncropped images for Fig.7B

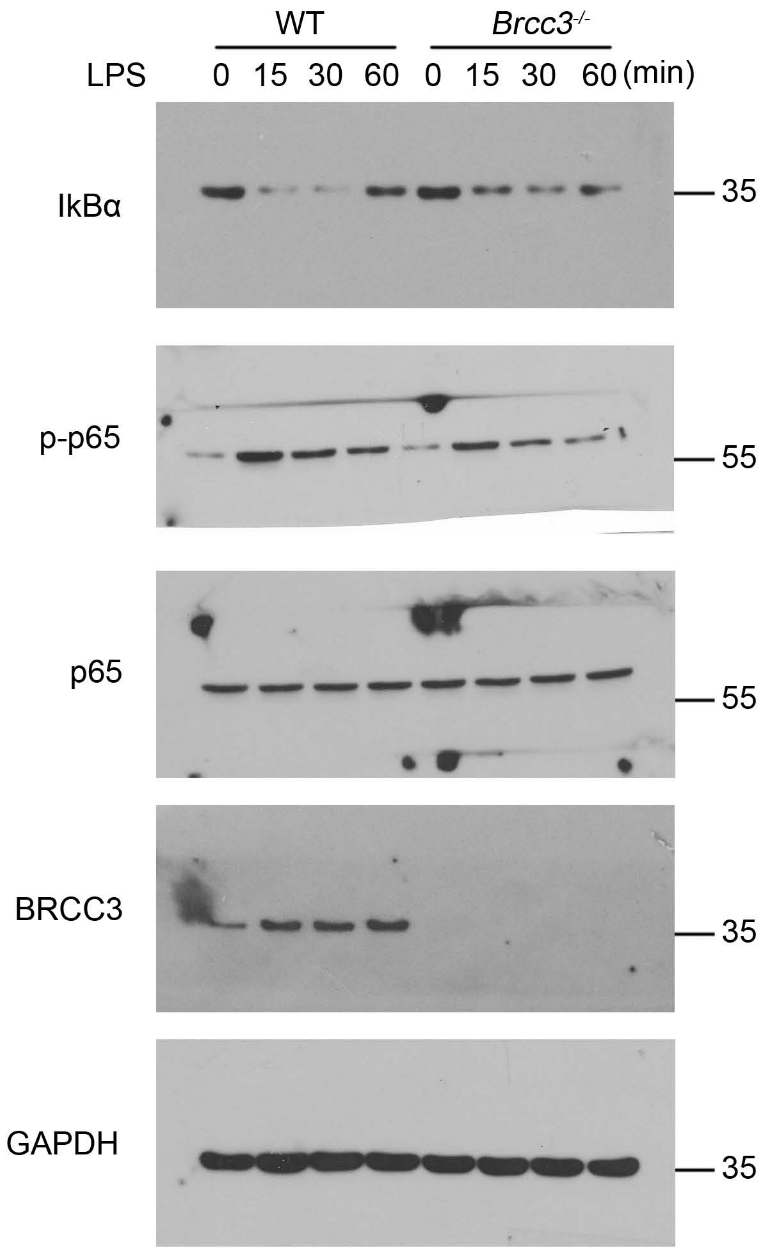

Uncropped images for Fig.7C PM

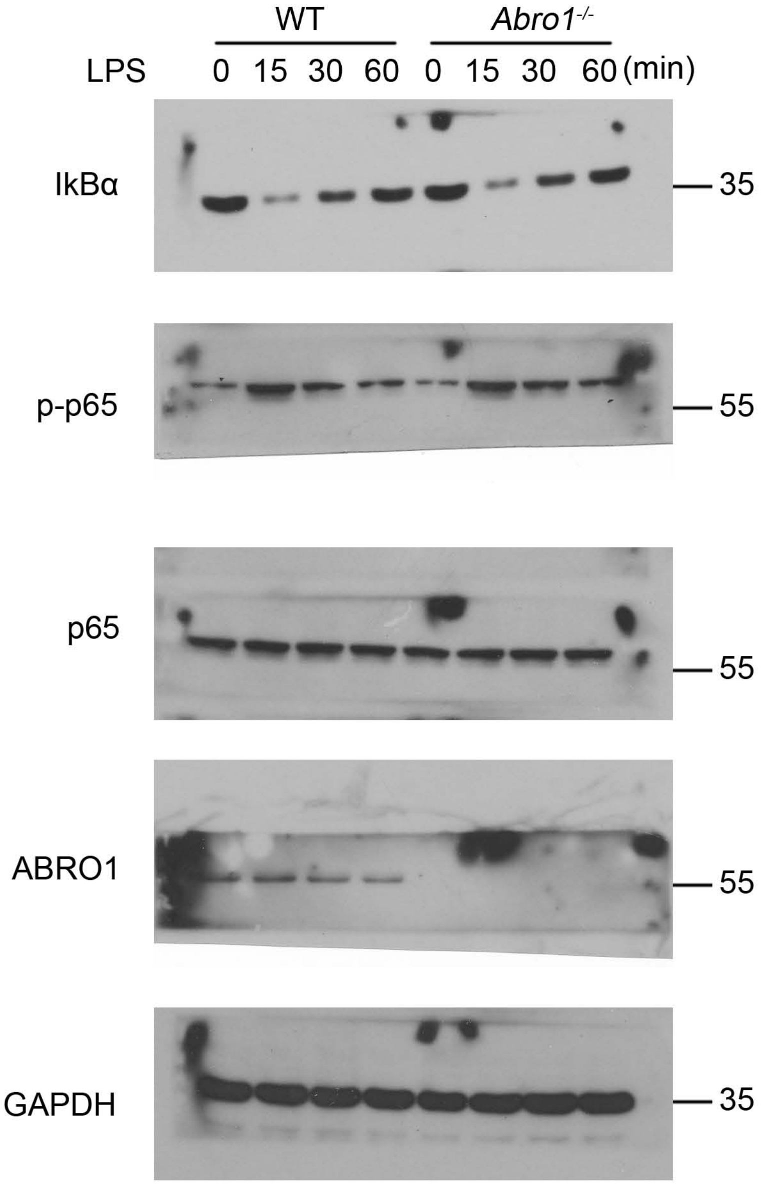

Uncropped images for Fig.7C NEUT

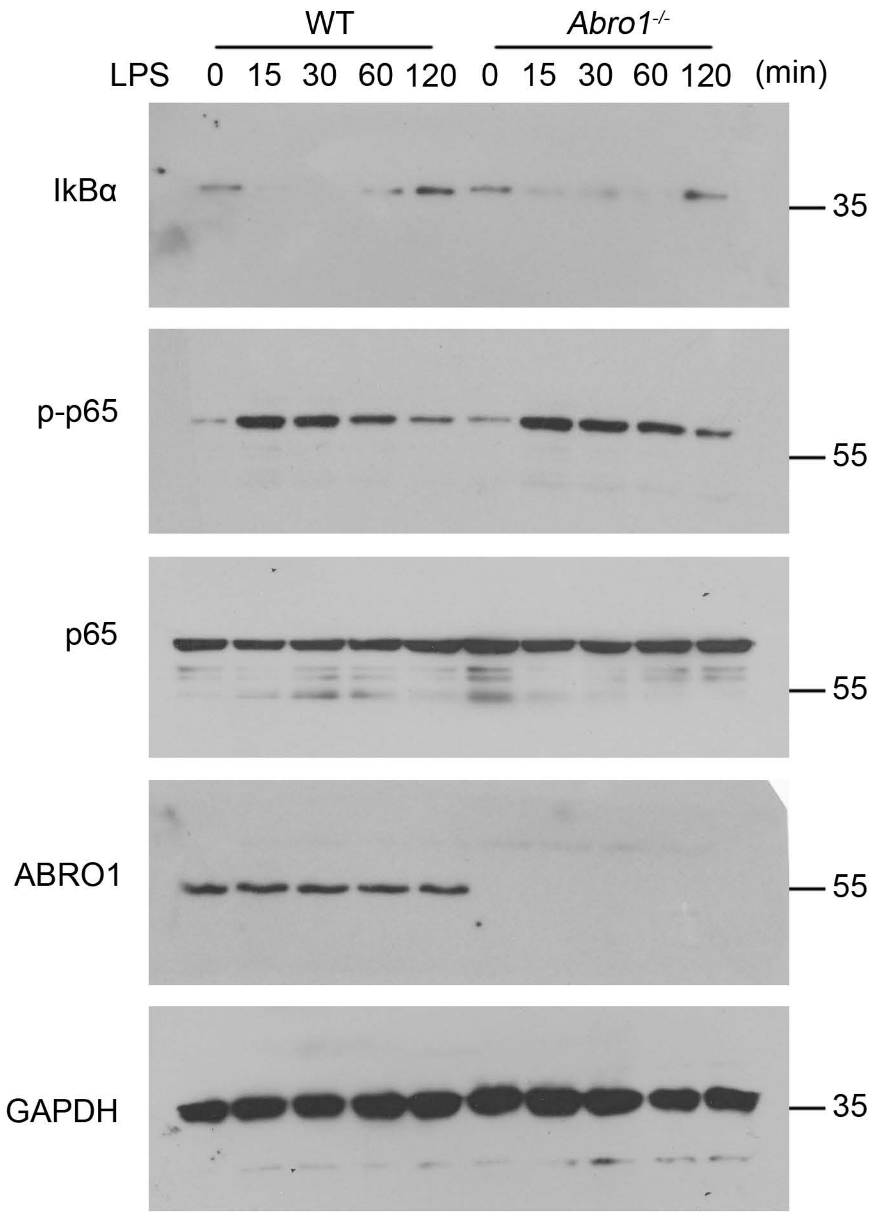

Uncropped images for Fig.7C BMDM

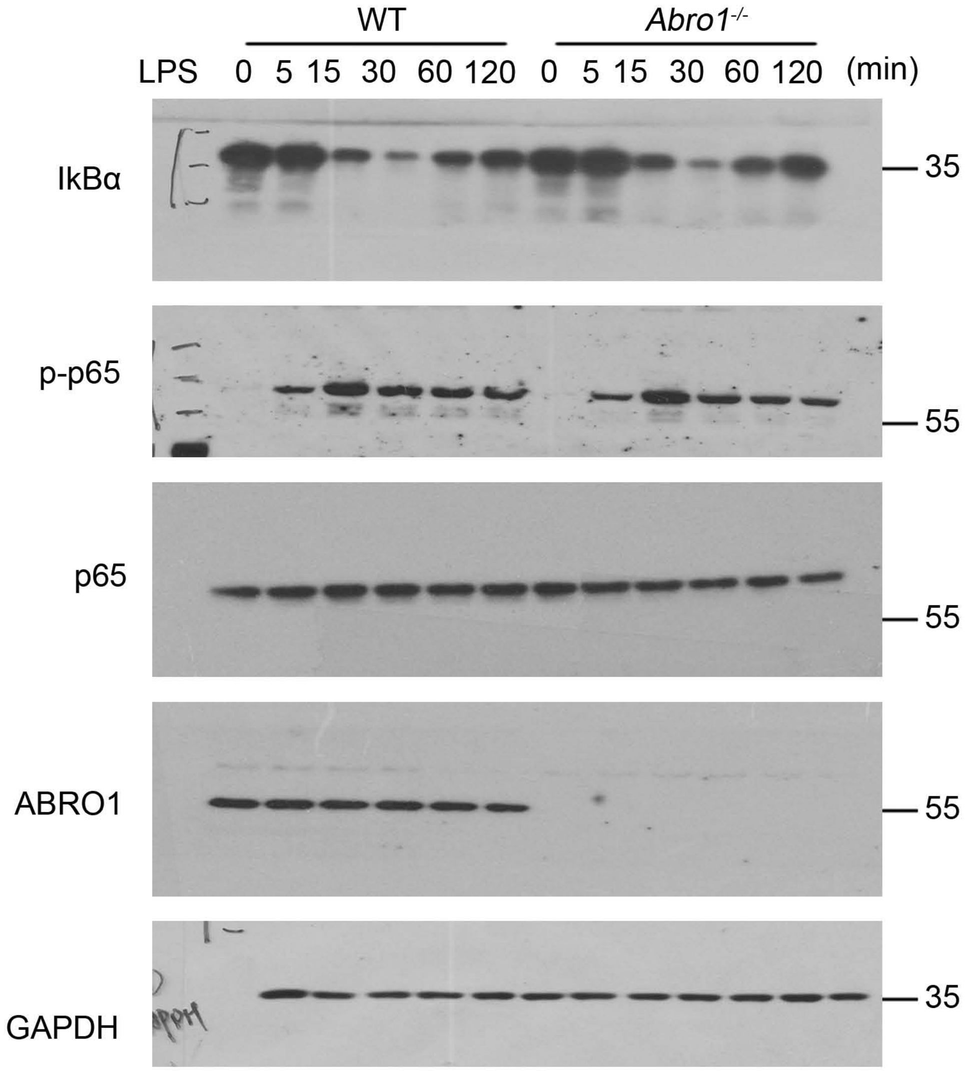

Uncropped images for Fig.7E

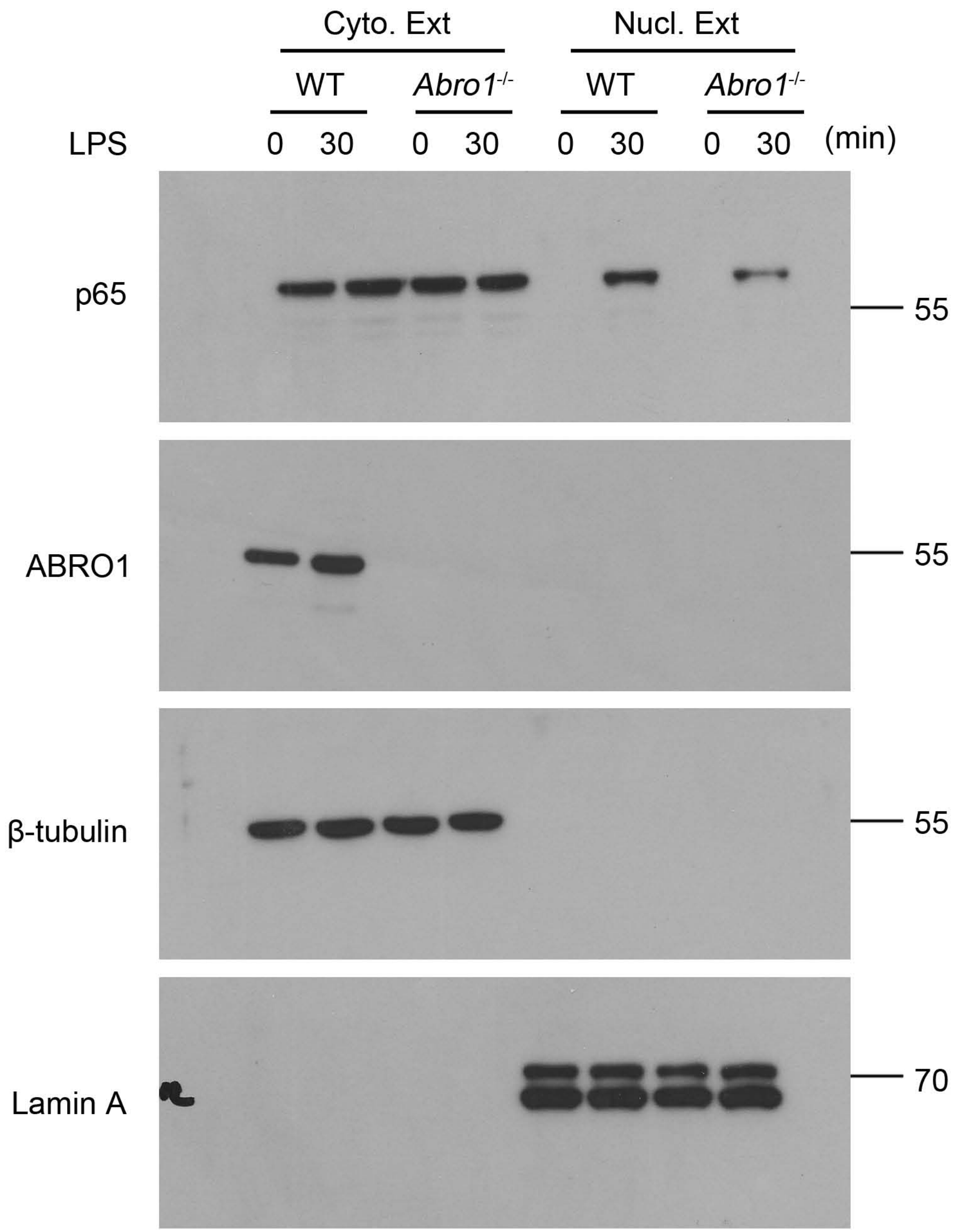

Uncropped images for Fig. S5B

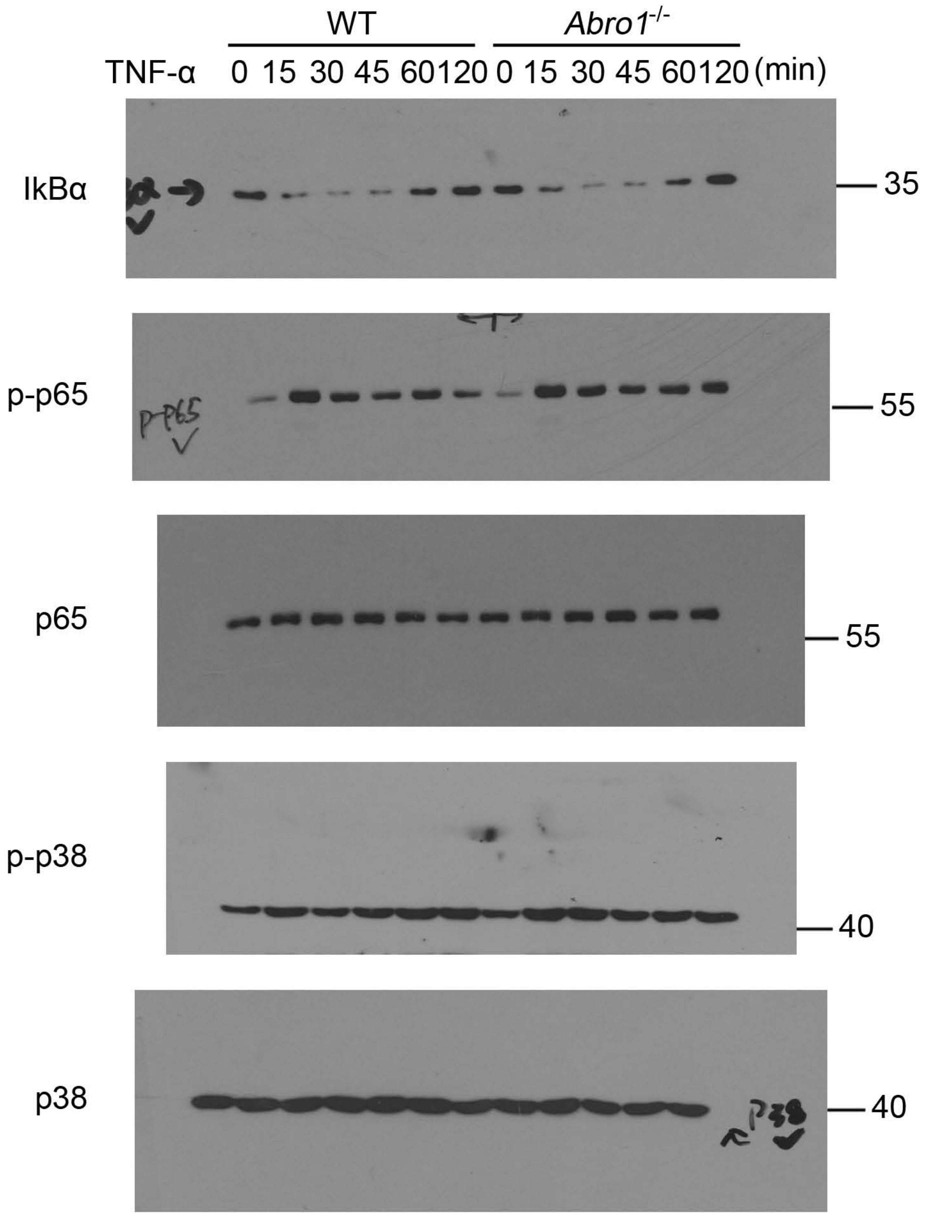

Uncropped images for Fig.S5B

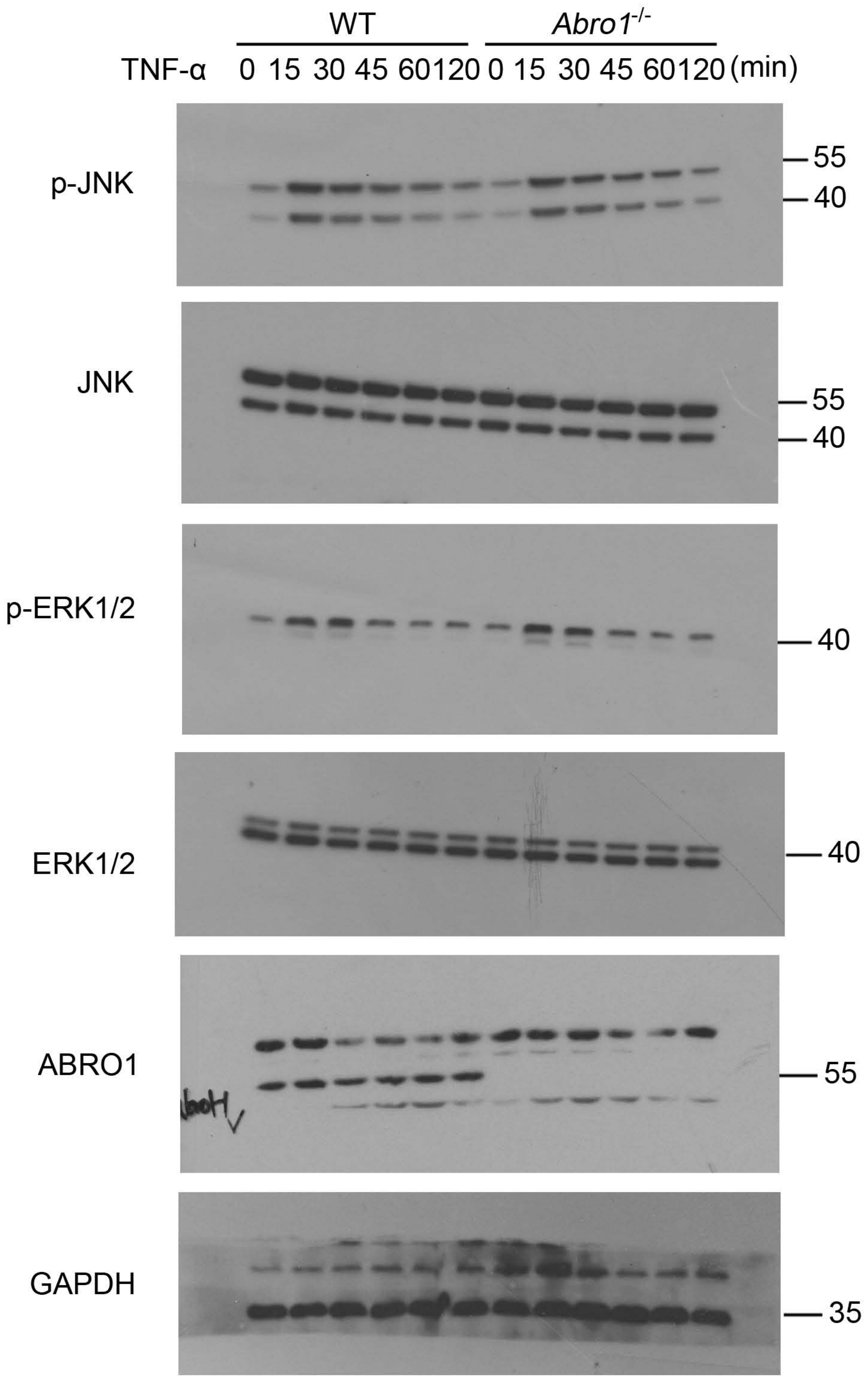

Uncropped images for Fig.S6 B-D

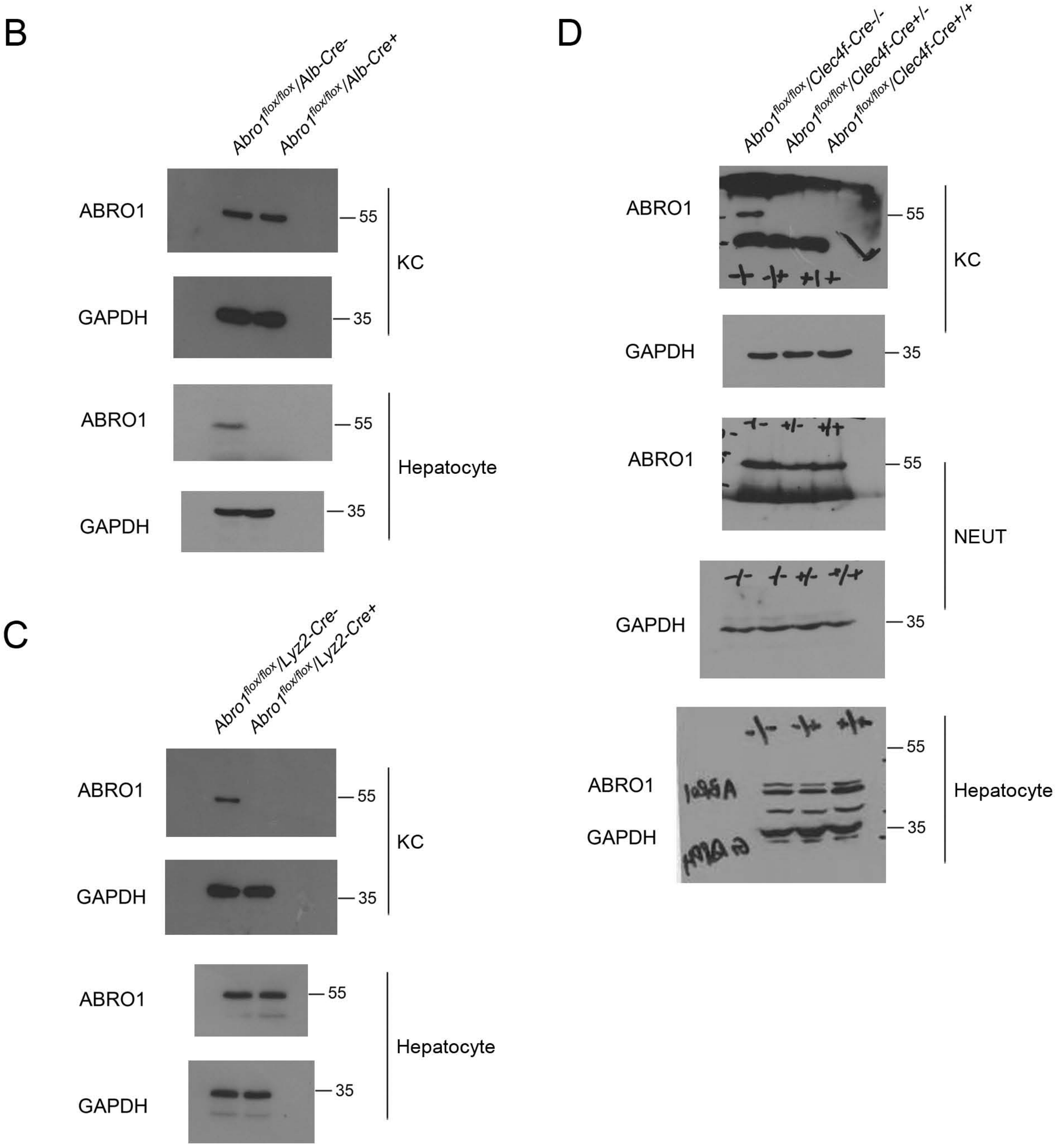

Uncropped images for Fig.S6 E

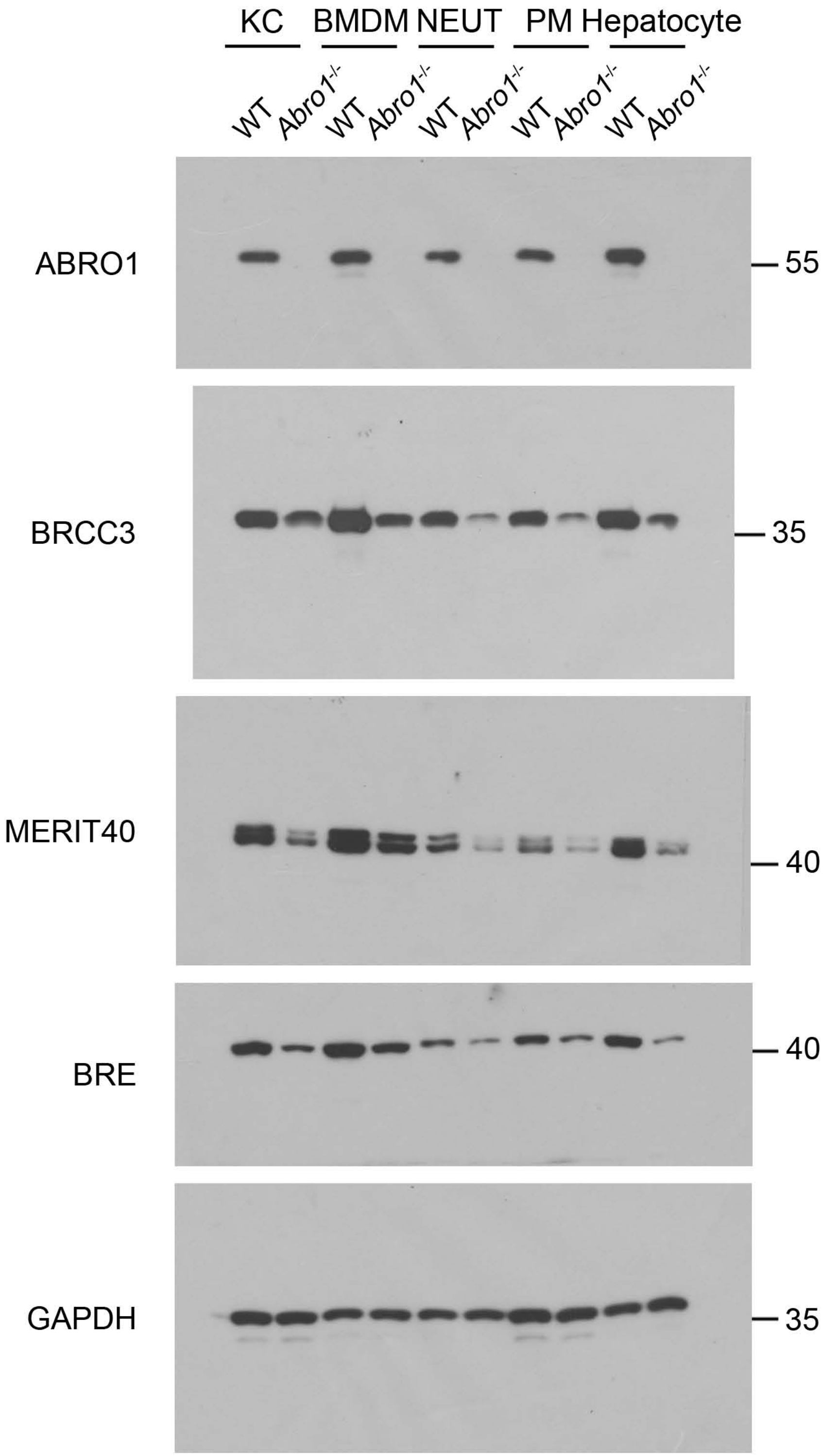

Supplement: Supplementary file 13 — Original Data File [file 41419_2023_6268_MOESM13_ESM.pdf]
